# Rapamycin-modified novel tolerogenic dendritic cells induce liver graft tolerance through MHC-II^+^CD8^+^ regulatory T cells

**DOI:** 10.1097/HC9.0000000000000942

**Published:** 2026-04-17

**Authors:** Lin Zhou, Yang Zhao, Jing Wang, Qing Chen, Ya-nan Jia, Li-chao Pan, Han-xuan Wang, Hong-wei Yang, Qiang He, Xian-liang Li, Ren Lang, Guo-sheng Du

**Affiliations:** 1 Division of Hepatobiliary and Pancreaticosplenic Surgery, Department of General Surgery, Beijing Chaoyang Hospital, Capital Medical University, Beijing, China; 2 Mass General Cancer Center, Mass General Brigham, Harvard Medical School, Somerville, Massachusetts, USA; 3 Department of General Surgery, Peking University Third Hospital, Beijing, China; 4 Faculty of Hepato-Pancreato-Biliary Surgery, Chinese PLA General Hospital, Beijing, China; 5 Organ Transplantation Center, General Hospital of Northern Theater Command, Shenyang, China

**Keywords:** liver transplantation, MHC-II^+^CD8^+^ Tregs, rapamycin, tolerogenic dendritic cells, trogocytosis

## Abstract

**Background:**

Inducing transplant tolerance to achieve long-term graft survival without immunosuppression remains a central objective in liver transplantation.

**Methods:**

Using a rat liver transplantation model, we evaluated the effects of infusing rapamycin-modified tolerogenic dendritic cells (Rapa-tolDCs) on graft survival and tolerance induction. Underlying molecular and cellular mechanisms were investigated through activation and inhibition experiments to assess the generation, signaling pathways, and suppressive functions of regulatory T and B cell populations.

**Results:**

Infusion of Rapa-tolDCs induced donor-specific tolerance and markedly prolonged graft survival (median survival time of 65 days, maximum of 102 days). Mechanistically, Rapa-tolDCs, characterized by low expression of Siglec1 and Spp1, functioned independently of the PI3K–mTOR pathway and promoted the differentiation and proliferation of CD8+CD45RClow/− regulatory T cells (CD8+CD45RClow/− Tregs). We identified that these Tregs acquired MHC-II molecules from donor cells via trogocytosis, becoming immune chimeric cells that predominantly secreted interleukin-10 (IL-10) to mediate immune suppression. The generation of these MHC-II+CD8+ Tregs was regulated by the Wnt5a/Fzd4/RhoD signaling axis. Furthermore, elevated levels of Foxp3+ Tregs and IL-10+ Bregs were found to contribute to prolonged graft survival following Rapa-tolDC infusion.

**Conclusions:**

These findings delineate a complete mechanistic pathway from cell therapy to donor-specific tolerance and provide a foundational strategy for clinical tolerance induction and post-transplant immunomodulation.

## HIGHLIGHTS

- Rapa-tolDCs induce transplant tolerance via MHC-II^+^CD8^+^ Tregs.
- Trogocytosis transfers MHC-II molecules from Rapa-tolDCs to CD8^+^ Tregs through the Wnt5a/Fzd4/RhoD signaling axis.
- MHC-II^+^CD8^+^ Tregs secrete IL-10 to mediate donor-specific immune suppression.

## INTRODUCTION

Only a small subset of carefully selected patients undergoing liver transplantation (LT) can maintain stable graft function even after discontinuation of all immunosuppressants (ISs) for more than 1 year.^1^ Approximately 20% of LT recipients may achieve complete freedom from ISs.^2^ However, most cases of induced immunotolerance remain unstable, and further verification is required to assess their clinical efficacy and safety.^3^,^4^,^5^ Achieving donor-specific tolerance through the use of regulatory cells represents an optimal strategy to overcome these limitations.

Tolerogenic dendritic cells (tolDCs), derived from bone marrow mesenchymal stem cells (BM-MSCs) or peripheral blood mononuclear cells, are promising regulatory cells for inducing immune tolerance.^6^ These cells are commonly generated using cytokines such as granulocyte–macrophage colony-stimulating factor (GM-CSF), genetic modification, or pharmacologic interventions involving rapamycin, vitamin D3 (VitD3), or 8-methoxypsoralen.^7^,^8^

Rapamycin precisely regulates immune cell differentiation via the mTOR pathway and demonstrates superior efficiency in inducing tolDCs. The phenotypic stability of rapamycin-induced tolDCs surpasses that of tolDCs induced by VitD3 and IL-10.^9^ Moreover, rapamycin enhances the immunomodulatory capacity of induced tolDCs, making it a promising approach for tolDC generation.^7^,^9^,^10^ Infusion of tolDCs has been shown to prolong graft survival in skin, liver, kidney, and heart transplantation models^7^,^8^,^11^,^12^,^13^ by promoting the induction of regulatory T cells (Tregs).^6^ Preliminary evidence regarding the safety and efficacy of single-dose tolDC infusion therapy in adult living-donor liver allograft recipients suggests that it may serve as a foundation for advancing immunotolerance research.^14^

The presence of CD8^+^CD45RC^low/−^ Tregs in LT recipients is positively correlated with stable graft function and inversely associated with acute rejection (AR),^15^,^16^,^17^ underscoring their crucial role in immune tolerance.^18^,^19^ Our previous studies demonstrated that induction of CD8^+^CD45RC^low/−^ Tregs extends graft survival in rat models of heart and liver transplantation following infusion of rapamycin-modified tolerogenic dendritic cells (Rapa-tolDCs) or plasmacytoid dendritic cells (pDCs).^18^,^19^,^20^,^21^,^22^ Notably, the expression of MHC-II molecules on CD8^+^CD45RC^low/−^ Tregs mediates donor-specific suppression, thereby contributing to graft survival.^19^ We further observed that trogocytosis-mediated acquisition of MHC-II molecules from pDCs generates MHC-II^+^CD8^+^CD45RC^low/−^ Tregs (MHC-II^+^CD8^+^ Tregs)—a novel chimeric Treg subset that exhibits donor-specific suppressive function and represents a potential strategy for inducing operational immune tolerance.^19^,^23^ Therefore, elucidating the mechanism by which MHC-II molecules are transferred to CD8^+^ Tregs via trogocytosis is essential for understanding donor-specific tolerance. We hypothesize that MHC-II molecules on the surface of Rapa-tolDCs can also be transferred through trogocytosis, leading to the generation of donor-specific MHC-II^+^CD8^+^ Tregs that promote long-term graft survival. This study aims to develop a feasible strategy for inducing clinical tolerance.

## METHODS

### Rats

All animal experiments were approved by the Animal Experiments and Experimental Animal Welfare Committee of Capital Medical University (Approval No. AEEI-2021-147; Approval Date: 2021-06-18). The procedures were performed in accordance with institutional guidelines for the care and use of laboratory animals, and the study is reported in accordance with the ARRIVE 2.0 guidelines. Male Brown Norway (BN, RT1n) and Lewis (RT1l) rats, aged 8–10 weeks and weighing 280–300 g, were obtained from Beijing Viton Lihua Animal Co., Ltd. (China). All animal procedures were approved by the Animal Experiments and Experimental Animal Welfare Committee of Capital Medical University (approval no. AEEI-2021-147). Animals were maintained under specific pathogen-free conditions in a controlled environment with a 12-hour light/dark cycle and were handled in compliance with the Animal Welfare Act and the Institutional Guidelines for the Care and Use of Laboratory Animals.

### Rat liver transplantation model

All animals were euthanized by intravenous injection of 10% potassium chloride under anesthesia. Combined inhalation and intravenous anesthesia was administered using isoflurane (RWD, China) at an induction flow rate of 0.8–1 L/h with a volatile concentration of 2%. Sodium pentobarbital (Sigma-Aldrich, USA) was administered intravenously at a concentration of 2% (0.2 mL/100 g body weight) for maintenance.

The rat LT model was established using the modified double-sleeve anastomosis technique.^18^,^22^

An AR model of LT was created by transplanting livers from Lewis donors into BN recipients, whereas spontaneous tolerance was modeled by transplanting livers from BN donors into Lewis recipients without any treatment or intervention.^24^,^25^,^26^

A total of 50 rats were randomly divided into 5 groups: the AR group (n=10), the tolerance (TOL) group (n=10), the Rapa-tolDCs group (n=15), the Rapa-imDCs group (n=15), and the blank control group (n=10).

### Rapa-tolDC preparation

A low-dose GM-CSF regimen was used to induce rRapa-tolDCs^22^,^27^. A detailed description of the experimental steps is provided in the Supplemental Methods, http://links.lww.com/HC9/C315.

The molecular and immunological characteristics of Rapa-tolDCs were analyzed using flow cytometry and transcriptomic techniques, as described in detail in the Supplemental Methods, http://links.lww.com/HC9/C315.

The ability of Rapa-tolDCs to stimulate effector T-cell proliferation was evaluated as described in the Supplemental Methods, http://links.lww.com/HC9/C315.

### Adoptive infusion regimen

A detailed description of the Rapa-tolDC infusion protocol is provided in the Supplemental Methods, http://links.lww.com/HC9/C315.

### Specimen acquisition

Details regarding sample collection at various time points are provided in the Supplemental Methods, http://links.lww.com/HC9/C315.

### Mixed lymphocyte culture

Rapa-tolDCs promoted the differentiation of CD8^+^CD45RC^low/−^ Tregs in mixed lymphocyte culture (MLC), as described in the Supplemental Methods, http://links.lww.com/HC9/C315. Interleukin-10 (IL-10) and interferon-γ (IFN-γ) stimulation protocols are also described therein.

The suppressive function of MHC-II^+^CD8^+^ Tregs induced by Rapa-tolDCs was assessed as described in the Supplemental Methods, http://links.lww.com/HC9/C315. The inhibitory effect was quantified based on the proliferation peak size of carboxyfluorescein succinimidyl ester (CFSE)-labeled CD4^+^CD25^−^ effector T cells, as reported by Li et al.^19^

### Laser confocal microscopy scanning

A detailed description of laser confocal microscopy procedures is provided in the Supplemental Methods, http://links.lww.com/HC9/C315.

### Signaling pathway analysis

A detailed description of the signaling pathways involved in MHC-II^+^CD8^+^ Treg generation is provided in the Supplemental Methods, http://links.lww.com/HC9/C315.

### Flow cytometry analysis

Detailed protocols for detecting CD8^+^CD45RC^low/−^ Tregs, MHC-II^+^CD8^+^ Tregs, IL-10, and IFN-γ expression are provided in the Supplemental Methods, http://links.lww.com/HC9/C315.

### HE staining and immunohistochemistry, protein isolation and western blotting, and multiplex immunofluorescence assay

Detailed descriptions of hematoxylin and eosin (HE) staining, immunohistochemistry (IHC), protein isolation, western blotting, and multiplex immunofluorescence assays are provided in the Supplemental Methods, http://links.lww.com/HC9/C315.

### Statistical analysis

Confocal images were acquired using a Leica TCS SP5 II system. All immunofluorescence and IHC images were analyzed with ImageJ software. Flow cytometry data were processed using FlowJo X, and all statistical analyses were performed with SPSS version 24.0. Data are presented as mean ± standard deviation (SD). Qualitative variables were compared using the *χ* ^2^ test, whereas quantitative variables were analyzed using one-way analysis of variance for multigroup comparisons or *t* tests for 2-group comparisons. Survival rates were calculated using the Kaplan–Meier method and evaluated using the log-rank test. Statistical significance was defined as *p*<0.05.

## RESULTS

### Low-dose GM-CSF combined with rapamycin modification as an effective approach for Rapa-tolDC preparation

**FIGURE 1:**
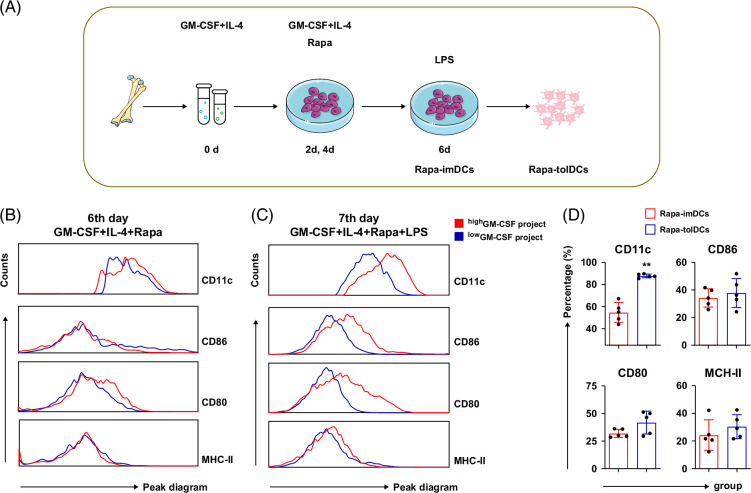
The schematic diagram of research and Rapa-tolDCs, the phenotypic differences of DCs derived from MSCs, which were stimulated by different GM-CSF regimens modified by rapamycin. The schematic representation of the experimental design for Rapa-tolDCs inducing (A). Both low and high concentrations of GM-CSF project induced MSCs-derived DCs expressed low levels of costimulatory molecules and MHC-II by modified with a small dose of rapamycin on the 6th day (B); after stimulation of LPS, DCs induced by low GM-CSF project still expressed low levels of costimulatory molecules and MHC-II (C). The DCs induced by low GM-CSF project at 6th and 7th day were named as Rapa-imDCs and Rapa-tolDCs, respectively. The Rapa-tolDCs were effectively resistant to LPS-stimulated maturation effects compared with Rapa-imDCs (D), which only showed a slight decrease in CD11c; n≥4 and **p＜0.01. Abbreviations: DCs, dendritic cells; GM-CSF, granulocyte–macrophage colony-stimulating factor; LPS, lipopolysaccharide; MHC-II, major histocompatibility complex class II; MSCs, mesenchymal stem cells; Rapa-imDCs, rapamycin-modified immature dendritic cells; Rapa-tolDCs, rapamycin-modified tolerogenic dendritic cells.

As shown in Figure 1A, MSC differentiation was induced using low or high concentrations of GM-CSF, while small doses of rapamycin were added on the second and fourth days to promote the generation of tolDCs. Dendritic cells (DCs) induced by the low-dose GM-CSF regimen exhibited lower surface expression of costimulatory molecules (CD80/CD86) and MHC-II compared with those induced by the high-dose regimen, with only a slight reduction in CD11c expression (Figures 1B, C). These features were consistent with the characteristics of tolDCs. Therefore, the low-dose GM-CSF regimen was selected as the standard protocol for Rapa-tolDC preparation in subsequent experiments.

DCs induced by the low-dose GM-CSF regimen that exhibited immature phenotypes on day 6 were designated as Rapa-imDCs (Figure 1D). These cells resisted lipopolysaccharide (LPS) stimulation and maintained stable immature characteristics, including low expression of costimulatory molecules (CD80/CD86) and MHC-II on day 7, and were termed Rapa-tolDCs. The expression of CD11c was higher in Rapa-tolDCs than in rapamycin-modified immature dendritic cells (Rapa-imDCs) (Figure 1D).

Additionally, immature DCs (imDCs) were generated using a high dose of GM-CSF without rapamycin modification (Supplemental Figure S1A, http://links.lww.com/HC9/C314) and subsequently differentiated into mature DCs (matDCs) following LPS stimulation. These matDCs exhibited high expression of MHC-II and costimulatory molecules, with a differentiation rate of CD11c^+^ DCs exceeding 95% (Supplemental Figure S1B, http://links.lww.com/HC9/C314). Collectively, these findings indicate that the combination of low-dose GM-CSF and rapamycin represents an effective method for the generation of Rapa-tolDCs.

### Rapa-tolDCs exhibit distinct transcriptional profiles with stable negative regulation and reduced lymphocyte-stimulatory and phagocytic capacities

Unlike the strong lymphocyte-proliferative capacity of matDCs, inactivated Rapa-tolDCs displayed a markedly weaker ability to stimulate lymphocyte proliferation, as evidenced by a significantly lower OD value at 450 nm and a reduced stimulation index (SI) (*p*<0.001) (Supplemental Table S1, http://links.lww.com/HC9/C314). In addition, Rapa-tolDCs demonstrated decreased phagocytic activity, reflected by a reduced uptake of fluorescein isothiocyanate-labeled dextran (*F*=26.7, *p*=0.0001) (Supplemental Figure S2, http://links.lww.com/HC9/C314) and diminished CD205 expression (*F*=36.05, *p*<0.0001) compared with Rapa-imDCs, imDCs, and matDCs (Supplemental Table S2, http://links.lww.com/HC9/C314). To further explore the transcriptional mechanisms underlying Rapa-tolDC–mediated tolerance, RNA sequencing was performed to compare gene expression between Rapa-tolDCs and matDCs. The heatmap of differentially expressed genes revealed 1176 downregulated genes in Rapa-tolDCs, with lighter color gradients indicating lower expression levels (Supplemental Figure S3A, http://links.lww.com/HC9/C314). Kyoto Encyclopedia of Genes and Genomes (KEGG) and Gene Set Enrichment Analysis (GSEA) pathway analyses indicated a pronounced downregulation of genes associated with the PI3K/Akt/mTOR signaling pathway in Rapa-tolDCs compared with matDCs (Supplemental Figures S3B–F, http://links.lww.com/HC9/C314). Protein–protein interaction (PPI) network analysis identified Siglec1 and Spp1 as transcriptionally regulated genes of potential biological significance (Supplemental Figure S4A, http://links.lww.com/HC9/C314). Preliminary validation confirmed that Siglec1 expression was low, while Spp1 expression progressively decreased, consistent with the mRNA sequencing results (Supplemental Figure S4B, http://links.lww.com/HC9/C314).

### Consecutive infusion of Rapa-tolDCs improves prognosis and survival in acute rejection rats, approaching levels observed in spontaneously tolerant recipients

**FIGURE 2:**
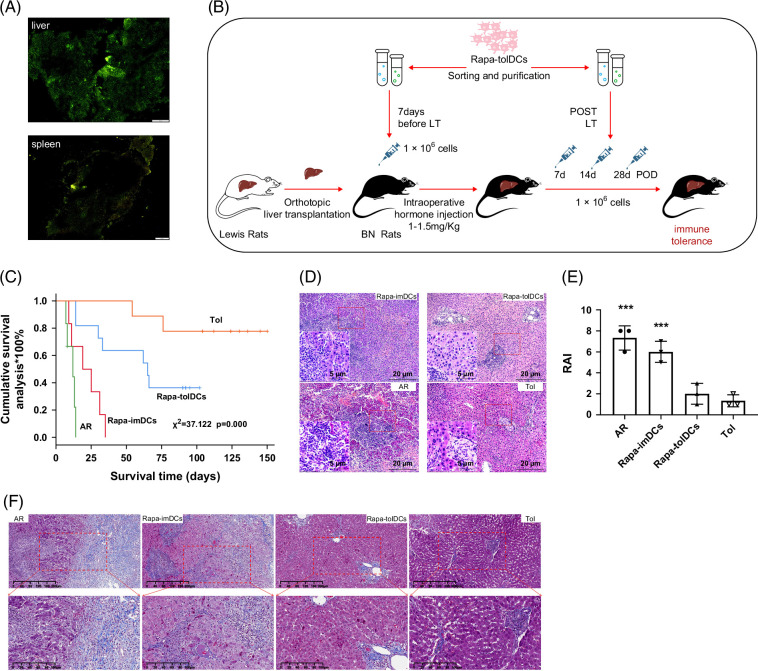
Survival, pathological results in different rats. Adoptively infused with CFSE-labeled Rapa-tolDCs into the AR rat of liver transplantation, and found that a strong fluorescence expression in the liver and spleen after 35 days of infusion (A). The schematic representation of the rat model and adoptive infusion (B). The adoptive infusion of Rapa-tolDCs promote immune tolerance by maintain long-term survival of liver graft (C), n≥6, and *p*=0.00; HE staining showed that the pathological features of liver graft of Rapa-tolDCs infused rats were basically consistent with those in rats with spontaneous immune tolerance (D) (image magnification, 100×/200×) n=6; RAI, a Banff diagnostic criteria for liver transplant rejection, was much lower in Rapa-tolDCs infusion rats when comparison with AR and Rapa-imDCs infusion rats, and had no difference with tolerance rats (E), n=3, and *** *p*<0.001. Masson trichome staining proved that the normal liver tissue in the AR rats was destroyed and replaced by fibrotic hyperplasia (F), while the fibrous staining in the Rapa-tolDCs re-fusion and tolerant rats was mainly concentrated in the blood vessels of the portal area, which was a normal change (image magnification, 100×, upper row/200×, lower row) n=4. Abbreviations: AR, acute rejection; CFSE, carboxyfluorescein succinimidyl ester; HE, hematoxylin–eosin; LT, liver transplantation; POD, postoperative day; RAI, rejection activity index; Rapa-imDCs, rapamycin-modified immature dendritic cells; Rapa-tolDCs, rapamycin-modified tolerogenic dendritic cells; Tol, tolerance.

To assess the persistence of Rapa-tolDCs in vivo, fluorescein-labeled Rapa-tolDCs were initially administered to liver-transplant rats. After 35 days, strong fluorescence signals were still detected in both the grafted liver and spleen, suggesting that Rapa-tolDCs may survive for extended periods or establish immune chimerism with host lymphocytes (Figure 2A). Following the Rapa-tolDC infusion protocol (Figure 2B), rats receiving Rapa-tolDCs exhibited markedly prolonged survival, with a median survival time (MST) of 65 days and a maximum survival time of 102 days. These durations were significantly longer than those of rats infused with Rapa-imDCs (MST, 19 d) and untreated AR rats (MST, 9 d) (Figure 2C). Histopathological examination using HE staining confirmed that grafted livers from Rapa-tolDC–treated rats exhibited nearly normal architecture, closely resembling the morphology observed in spontaneously tolerant (sTOL) rats. In contrast, livers from Rapa-imDC–infused and AR rats displayed marked structural damage (Figure 2D). The hepatic lobular structures of Rapa-tolDC–treated rats remained largely intact, showing only mild inflammatory responses and limited lymphocyte infiltration localized mainly in the portal regions (Figure 2D). The rejection severity, quantified by the Rejection Activity Index (RAI), was significantly higher in AR and Rapa-imDC–treated than in Rapa-tolDC–treated and sTOL rats (Figure 2E). Furthermore, the Masson trichrome staining revealed that liver tissues from Rapa-tolDC–treated and spontaneously tolerant rats exhibited near-normal histological features, whereas those from AR rats showed evident fibrotic alterations (Figure 2F).

### Elevated levels of MHC-II^+^CD8^+^ Tregs and IL-10 contribute to prolonged survival in acute rejection rats following consecutive infusion of Rapa-tolDCs

**FIGURE 3:**
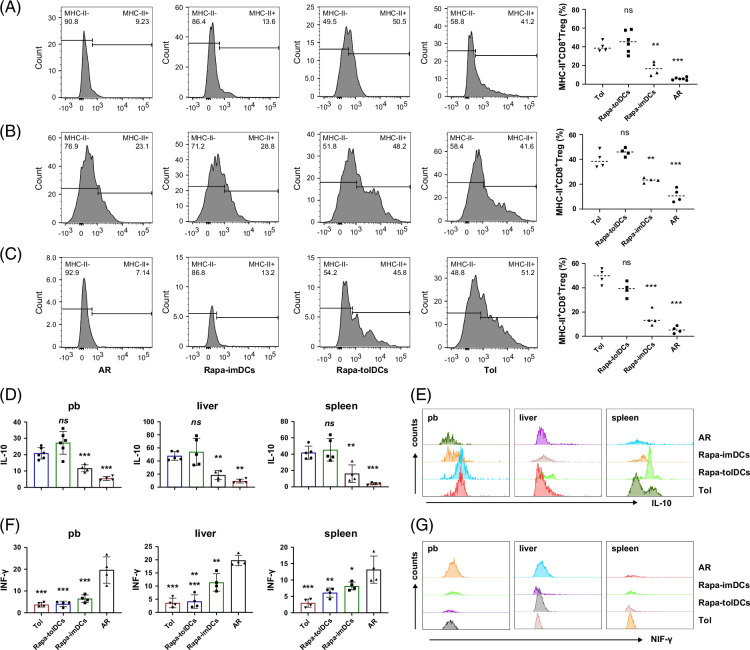
The expression difference of MHC-II^+^CD8^+^ Tregs and their secreted IL-10 and INF-γ in rats after liver transplantation. The levels of MHC-II^+^CD8^+^ Tregs in peripheral blood (A, n≥4), transplanted liver (B, n=4), and spleen (C, n=4) of rats with Rapa-tolDCs adoptive infusion were higher than those of AR and Rapa-imDCs adoptive infusion rats, and there was no difference between Rapa-tolDCs adoptive infusion rats and Tol rats. (D–G) The IL-10 and INF-γ of MHC-II^+^CD8^+^ Tregs in different tissues of liver transplantation rats were identified by flow cytometry. (D) The MHC-II^+^CD8^+^ Treg levels of IL-10 secreted in peripheral blood, transplanted liver, and spleen of Rapa-tolDC adoptive infusion rats were higher than those of AR and Rapa-imDCs adoptive infusion rats, and there was no difference between Rapa-tolDCs adoptive infusion rats and tolerance rats; ***p＜0.001, **p＜0.01, *p<0.05, and NS, no difference. (E) A representative peak image by flow cytometry. (F) The MHC-II^+^CD8^+^ Treg levels of INF-γ secreted in peripheral blood, transplanted liver, and spleen of Rapa-tolDCs and Rapa-imDCs adoptive infusion rats and tolerance rats were lower than those of AR rats, and there was no difference between Rapa-tolDCs adoptive infusion rats and tolerance rats; ***p＜0.001, **p＜0.01, *p<0.05, and ns, no difference. (G) A representative peak image by flow cytometry. Rapa-tolDCs and Rapa-imDCs liver transplantation were significantly different. Abbreviations: AR, acute rejection; IFN-γ, interferon-γ; IL-10, interleukin-10; MHC-II^+^CD8^+^ Tregs, MHC class II–positive CD8^+^ regulatory T cells; Rapa-imDCs, rapamycin-modified immature dendritic cells; Rapa-tolDCs, rapamycin-modified tolerogenic dendritic cells; Tol, tolerance (tolerant rats).

The frequency of MHC-II^+^CD8^+^ Tregs and their cytokine secretion profiles in different tissues, as determined by flow cytometry, were used as indicators of immune tolerance following Rapa-tolDC infusion. The gating strategy applied for flow cytometric analysis is described in detail in the Supplemental Materials, http://links.lww.com/HC9/C314. MHC-II^+^CD8^+^ Tregs were significantly elevated in the peripheral blood, grafted liver, and spleen of rats treated with Rapa-tolDCs compared with those in AR rats and rats receiving Rapa-imDCs (*p*<0.01 for all comparisons) (Figures 3A–C and Supplemental Figure S5A, http://links.lww.com/HC9/C314). Furthermore, MHC-II^+^CD8^+^ Tregs from the peripheral blood, transplanted liver, and spleen of Rapa-tolDC–treated rats secreted higher levels of interleukin-10 (IL-10) (Figures 3D, E and Supplemental Figure S5A, http://links.lww.com/HC9/C314) and lower levels of INF-γ (Figures 3F, G) relative to the other groups. In contrast, AR and Rapa-imDC–treated rats exhibited the opposite cytokine expression pattern. Notably, IFN-γ levels differed significantly only in the grafted liver between Rapa-tolDC–treated and Rapa-imDC–treated rats (*p*<0.01). No significant differences were observed between Rapa-tolDC–treated rats and sTOL rats in these immunological parameters, suggesting that the consecutive infusion of Rapa-tolDCs induces tolerance through the generation of MHC-II^+^CD8^+^ Tregs and enhanced IL-10 secretion.

### MHC-II^+^CD8^+^ Tregs, Foxp3^+^ Tregs, and IL-10^+^ Bregs in liver grafts are crucial for immunotolerance following consecutive infusion of Rapa-tolDCs

**FIGURE 4:**
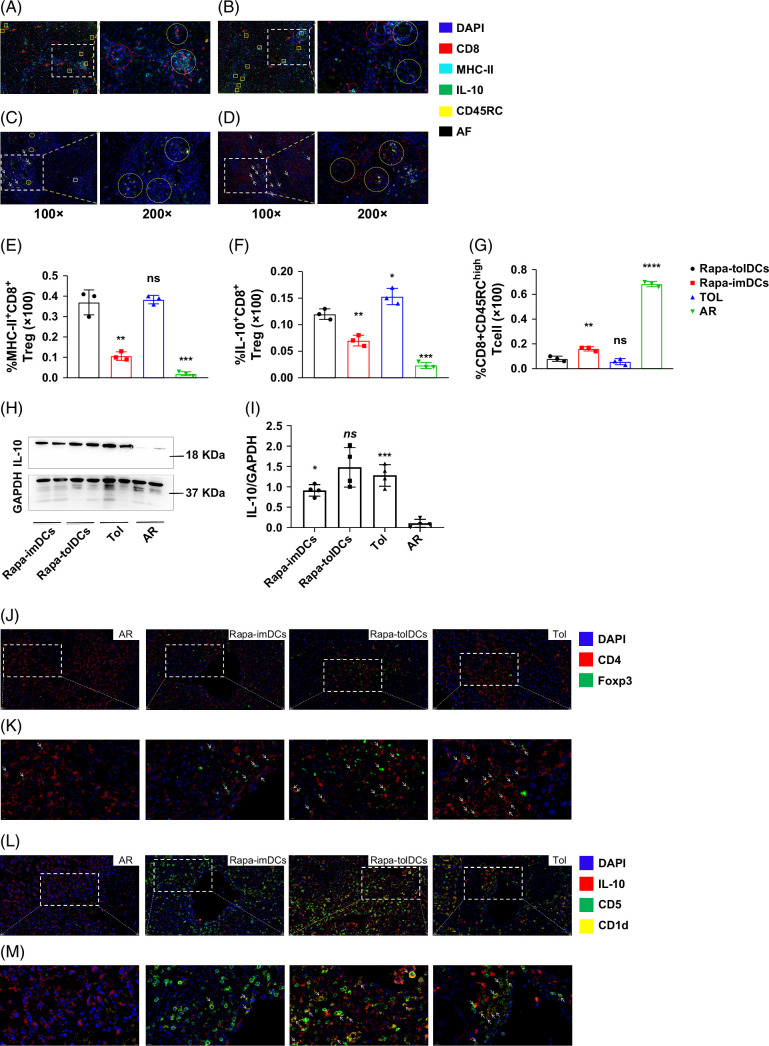
Multicolor immunofluorescence staining (mIFs) results in different rat models indicated high levels of MHC-II^+^CD8^+^CD45RC^low/−^ Tregs, Foxp3^+^ Tregs, and IL-10^+^ Bregs in the transplanted liver of Rapa-tolDCs adoptive infusion rats and in spontaneous immune tolerance rats. To confirm that MHC-II^+^CD8^+^ Treg is the main cell subgroup that induces immune tolerance, mIFs were used to identify the CD8, CD45RC, MHC-II, and IL-10 in different recipients (A–D). The MHC-II^+^CD8^+^CD45RC^low/−^ Treg (highlighted with yellow box in 100× image and yellow circle in 200× image) was highly expressed in spontaneous tolerance (A) and Rapa-tolDCs infusion (B) rat of liver transplantation, and the MHC-II^+^CD8^+^CD45RC^low/−^ Treg, which secreted IL-10 (red arrow in 100× and red circle in 200×) was also highly expressed in these 2 groups (A, B). Although the IL-10 (yellow ellipse in 100×) was expressed in Rapa-imDCs infusion (C) and AR (D) rats, not expressed in the MHC-II^+^CD8^+^CD45RC^low/−^ Treg. The AR rats only expressed effector T cells with high expression of CD45RC (white arrow in 100× and yellow circle in 200×) (D). Each group has at least n≥4, with a total of n=20 for the mIF staining. (E–G) A high level of MHC-II^+^CD8^+^ Tregs (E) and IL-10^+^CD8^+^ Tregs (F) in Rapa-tolDCs infusion rats versus spontaneous tolerance rats, Rapa-imDCs infusion rats, and AR rats. Moreover, an obviously high level of CD8^+^CD45RC^high^ T cells in AR rats than in others (G); ***p<0.001, **p<0.01, *p<0.05, and ns, no difference. Three representative samples of each group were selected for statistical analysis. At least 3 sections were counted for each sample, and at least 6 independent fields were analyzed for each section. (H, I) Protein expression levels of IL-10 in the transplanted liver of different rats. (H) Western blot results showed that the expression of IL-10 in the transplanted liver of Rapa-tolDCs infusion rats was higher than that of Rapa-imDCs infusion rats, and AR rats' full-length blots/gels were presented in Supplemental Figure S6, http://links.lww.com/HC9/C314. (I) One-way ANOVA showed that there was no difference in IL-10 expression between Rapa-tolDCs adoptive infusion rats and spontaneously tolerant rats, which was significantly higher than that in Rapa-imDCs adoptive infusion rats and AR rats, n=4, ***p<0.001, *p<0.05, and ns, no difference. (J–M) mIFs also indicated high numbers of CD4^+^Foxp3^+^ Tregs (J, K) and IL-10^+^Breg (L, M) in Rapa-tolDCs infusion rats than in AR rats and Rapa-imDCs infusion rats. (J) and (L) were 100× field; (K) and (M) were 200× field. mIF staining n=4 for each group, with a total n=20, 3 representative samples of each group were selected for statistical analysis. At least 3 sections were counted for each sample, and at least 6 independent fields were analyzed for each section. Abbreviations: ANOVA, analysis of variance; AR, acute rejection; Bregs, regulatory B cells; DCs, dendritic cells; Foxp3, forkhead box P3; IFN-γ, interferon-γ; IL-10, interleukin-10; mIFs, multicolor immunofluorescence staining; MHC-II, major histocompatibility complex class II; Rapa-imDCs, rapamycin-modified immature dendritic cells; Rapa-tolDCs, rapamycin-modified tolerogenic dendritic cells; Tol, spontaneous immune tolerance rats; Tregs, regulatory T cells.

Compared with rats infused with Rapa-imDCs, those treated with Rapa-tolDCs or exhibiting sTOL displayed strong fluorescence signals for CD8, MHC-II, and IL-10, while CD45RC expression was weak or undetectable in liver grafts (Figures 4A–C). In contrast, liver grafts from AR rats showed marked lymphocyte infiltration with intense fluorescence for CD8 and CD45RC (Figure 4D). Multiplex immunofluorescence (mIF) analysis further revealed higher expression of MHC-II^+^CD8^+^ Tregs secreting IL-10 in rats treated with Rapa-tolDCs and in sTOL rats compared with those treated with Rapa-imDCs or AR rats (Figures 4E, F).

Moreover, the high expression of CD8^+^CD45RC^high^ T cells in AR rats was accompanied by minimal IL-10 secretion, indicating severe inflammation and tissue injury (Figures 2D–F, 4F, G). Western blot analysis also demonstrated significantly higher IL-10 protein levels in Rapa-tolDC–treated and sTOL rats than in AR and Rapa-imDC–treated rats (Figures 4H, I and Supplemental Figure S6, http://links.lww.com/HC9/C314). Collectively, these findings indicate that MHC-II^+^CD8^+^ Tregs are closely associated with immune tolerance induced by consecutive Rapa-tolDC infusion following liver transplantation.

Additionally, mIF results showed that expression of CD4, Foxp3, IL-10, CD5, and CD1d in the transplanted livers of Rapa-tolDC–treated rats was strongly positive (Figures 4J–M), exceeding the levels observed in AR and Rapa-imDC–treated rats. This finding further confirms that Rapa-tolDC infusion enhances the expression of CD4^+^Foxp3^+^ Tregs and IL-10^+^ Bregs, highlighting the multifaceted immunoregulatory mechanisms underlying Rapa-tolDC–induced tolerance.

### Rapa-tolDCs promote the generation of IL-10^+^CD8^+^CD45RC^low/−^ Tregs

**FIGURE 5:**
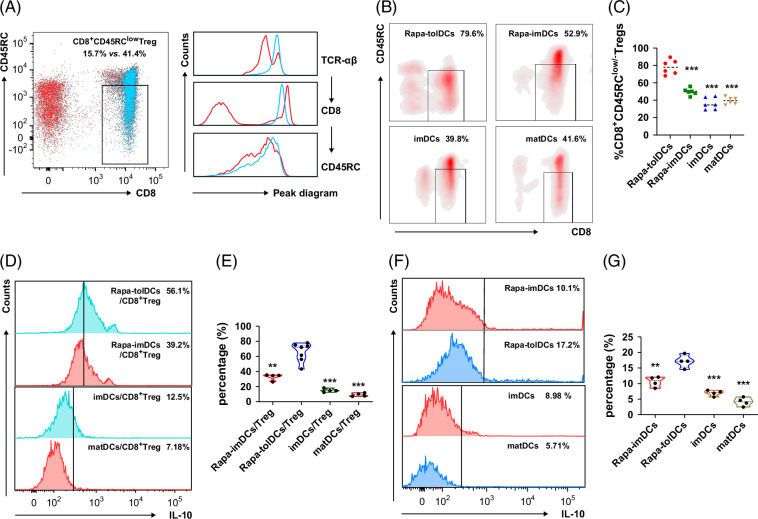
Rapa-tolDCs induce differentiation and production of CD8^+^CD45RC^low/−^ Tregs with high IL-10 secretion. The level of CD8^+^CD45RC^low/−^ Tregs in the spleen tissues of normal rats is about 15%–20%, and the proportion after sorting is about 40% (A), scatter plot and peak plot all identified by flow cytometry. When co-cultured with Rapa-tolDCs, the expression level of CD8^+^CD45RC^low/−^ Tregs was promoted compared with that with Rapa-imDCs, imDCs, and mature DCs (B), CD8^+^CD45RC^low/−^ Tregs identified by flow cytometry; the ANOVA analysis indicated an obvious difference in different groups (C); n=6 and ***p＜0.001 versus Rapa-tolDCs. Peak plots all identified by flow cytometry, which indicating that both DCs and CD8^+^CD45RC^low/−^ Tregs secreted IL-10, but a high level of IL-10 were detection from the mixture of CD8^+^CD45RC^low/−^ Tregs (D), with an obvious difference of Rapa-tolDCs/CD8^+^CD45RC^low/−^ Tregs when comparison with others (E); n≥4 and a rather low level of DCs for each group (F), with difference in Rapa-tolDCs determined by one-way ANOVA (G), n≥4 when comparison with others. **p＜0.01 and ***p＜0.001. Abbreviations: ANOVA, analysis of variance; DCs, dendritic cells; IL-10, interleukin-10; imDCs, immature dendritic cells; Rapa-imDCs, rapamycin-modified immature dendritic cells; Rapa-tolDCs, rapamycin-modified tolerogenic dendritic cells; TCR, T-cell receptor; Tregs, regulatory T cells.

We first demonstrated that Rapa-tolDCs induce the differentiation of naïve CD8^+^ T cells into CD8^+^CD45RC^low/−^ Tregs (Figures 5A, B). The proportion of CD8^+^CD45RC^low/−^ Tregs in the spleens of normal rats was 15.27±1.89%, which increased to 42.06±2.24% following magnetic bead sorting (Figure 5A). After co-culture with Rapa-tolDCs, the percentage of CD8^+^CD45RC^low/−^ Tregs was further upregulated to ~80%, significantly higher than that observed with Rapa-imDCs, imDCs, or matDCs (Figures 5B, C). The levels of IL-10 in mixed lymphocyte cultures were subsequently assessed. All DC types increased IL-10 secretion upon co-culture; however, Rapa-tolDCs induced the highest levels (Figures 5D–F). Statistical analysis confirmed that both the frequency of CD8^+^CD45RC^low/−^ Tregs and IL-10 secretion were significantly greater in the Rapa-tolDC group than in the others (*p*<0.01) (Figures 5C, E–G). Although Rapa-tolDCs themselves secreted IL-10, the secretion was comparatively modest, whereas CD8^+^CD45RC^low/−^ Tregs were the dominant source of IL-10 (Figures 5E–G). These findings suggest that IL-10 production constitutes a key mechanism underlying the immunosuppressive function of CD8^+^CD45RC^low/−^ Tregs.

### MHC-II^+^CD8^+^ Tregs with donor-specific inhibitory properties represent the primary functional subset of CD8^+^CD45RC^low/−^ Tregs induced by Rapa-tolDCs

**FIGURE 6:**
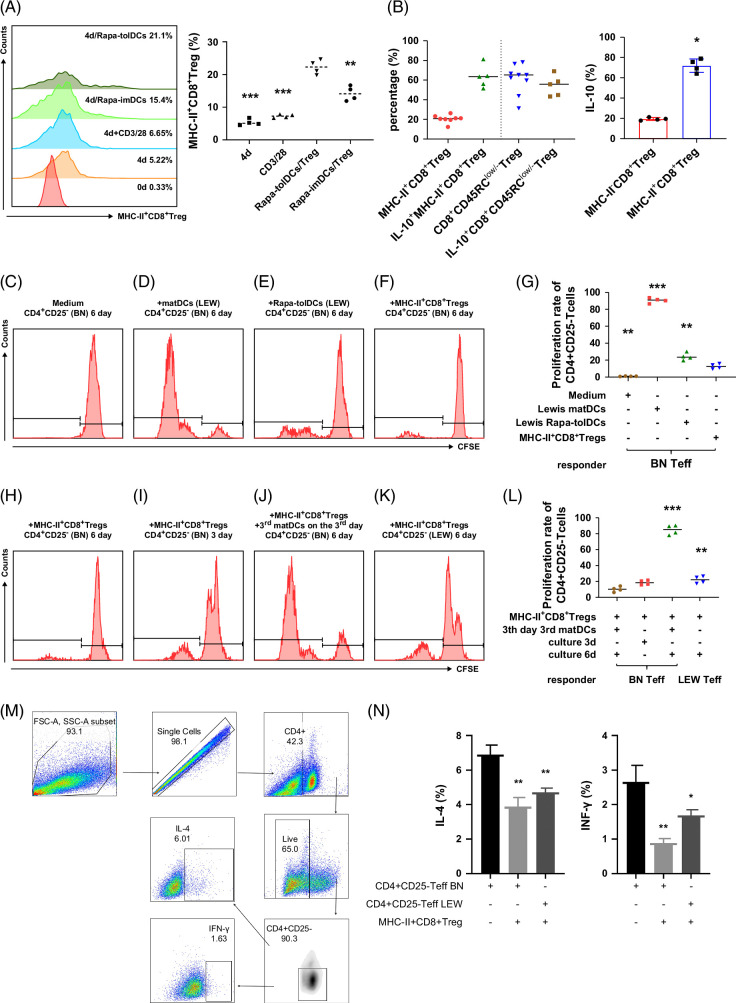
The MHC-II^+^CD8^+^ Tregs induced by Rapa-tolDCs, which may be the main functional subgroup of CD8^+^CD45RC^low/−^ Tregs plays a donor-specific suppression on the proliferation of CD4^+^CD25^−^ T cells. The CD8^+^T cells cannot be effectively differentiated into CD8^+^CD45RC^low/−^ Tregs with only supplement of CD3 and CD28 (A), but a higher differentiation rates when CD8^+^T cells co-cultured with Rapa-tolDCs and Rapa-imDCs; the difference of CD8^+^CD45RC^low/−^ Tregs induced by Rapa-tolDCs was significantly comparison with Rapa-imDCs and others determined by one-way ANOVA (A), n=5 independently repetition; **p＜0.01 and ***p＜0.001. Although the percentage of MHC-II^+^CD8^+^ Tregs was only about 20%, but the IL-10 secreted level of which occupy the dominating part according to one-way ANOVA (B), n=5 independently repetition,*p＜0.05; furthermore, the levels of IL-10 secreted from subpopulation of MHC-II positive of CD8^+^CD45RC^low/−^ Tregs is about 4-folds as large as the MHC-II negative subpopulation according to one-way ANOVA (B), n=4. MHC-II^+^CD8^+^ Tregs were used as stimulation cells, and CD4^+^CD25^-^Teff were used as responder cells to perform a donor-specific inhibition experiment of proliferation (C–L). The proliferation of CD4^+^CD25^−^ T cells was significantly enhanced when mature DCs from Lewis rat was added (C, D); however, when Rapa-tolDCs and MHC-II^+^CD8^+^ Tregs added, it showed a much higher inhibitory effect to CD4^+^CD25^−^ T-cell proliferation of MHC-II^+^CD8^+^ Tregs according to one-way ANOVA (E–G), n=4 independently repetition for each experiments; **p＜0.01 and ***p＜0.001. The inhibitory effect on the proliferation of CD4^+^CD25^−^ T cell of MHC-II^+^CD8^+^ Tregs were enhanced with time (3 d vs. 6 d) (H, I) and that were much higher in homogeneous (BN) than in heterogeneous (LEW) (H, K), and when the third mature DCs was added, its inhibitory effect on proliferation was weakened or canceled determined by one-way ANOVA (J, L); n=4 independently repetition for each experiments, **p＜0.01, and ***p＜0.001. After co-culturing CD4^+^CD25^−^ T cells with MHC-II^+^CD8^+^ Tregs for 48 hours, flow cytometry was used to detect the levels of IL-4 and INF-γ (M). It was found that the ability of CD4^+^CD25^−^ T cells to secrete Th1 and Th2 cytokines was weakened (N), and this effect was more pronounced in the same strain (N). n≥4 independent repetitions for each experiment according to one-way ANOVA, *p＜0.05, and **p＜0.01. Abbreviations: ANOVA, analysis of variance; DCs, dendritic cells; IL-4, interleukin-4; IL-10, interleukin-10; IFN-γ, interferon-γ; MHC-II, major histocompatibility complex class II; Rapa-imDCs, rapamycin-modified immature dendritic cells; Rapa-tolDCs, rapamycin-modified tolerogenic dendritic cells; Teff, effector T cells; Tregs, regulatory T cells.

Our findings demonstrated that the proportion of CD8^+^CD45RC^low/−^ Tregs expressing high levels of MHC-II was significantly greater in the Rapa-tolDC group than in the Rapa-imDC and control groups (Figure 6A). Analysis of IL-10 production among subpopulations of CD8^+^CD45RC^low/−^ Tregs revealed that MHC-II–positive CD8^+^CD45RC^low/−^ Tregs comprised ~20%–25% of the total CD8^+^CD45RC^low/−^ Treg population yet accounted for nearly 80% of total IL-10 secretion (Figure 6B). These results indicate that the MHC-II–high subset represents the principal source of IL-10 production within the CD8^+^CD45RC^low/−^ Treg population, suggesting its potential role in mediating donor-specific immunosuppressive effects.

In the CFSE cell proliferation assay, MHC-II^+^CD8^+^ Tregs were generated from co-cultures of donor-derived Rapa-tolDCs (LEW) and recipient CD8^+^ Tregs (BN). These MHC-II^+^CD8^+^ Tregs were subsequently cultured separately with syngeneic (BN) or allogeneic (LEW) CD4^+^CD25^−^ effector T cells. Compared with the control and matDC groups (Figures 6C, D), both Rapa-tolDCs (Figure 6E) and MHC-II^+^CD8^+^ Tregs (Figure 6F) exhibited a markedly reduced capacity to stimulate CD4^+^CD25^−^ effector T-cell proliferation (*p*<0.01) (Figure 6G). These results indicate that MHC-II^+^CD8^+^ Tregs possess potent inhibitory activity against CD4^+^CD25^−^ effector T-cell proliferation. The suppressive effect of MHC-II^+^CD8^+^ Tregs on CD4^+^CD25^−^ effector T-cell proliferation in syngeneic rats became progressively more pronounced over time (Figures 6H, I, L). Addition of matDCs from third-party donors attenuated the proliferation of CD4^+^CD25^−^ effector T cells in syngeneic cultures (Figure 6J, L), suggesting that the inhibitory function of MHC-II^+^CD8^+^ Tregs is donor-specific. Moreover, inhibition of CD4^+^CD25^−^ effector T-cell proliferation by MHC-II^+^CD8^+^ Tregs was significantly stronger in syngeneic cultures than in allogeneic ones (Figures 6K, L).

Further analysis revealed that expression levels of interleukin-4 (IL-4) and INF-γ in CD4^+^CD25^−^ effector T cells were significantly reduced after 48 hours of co-culture with MHC-II^+^CD8^+^ Tregs compared with controls, suggesting that MHC-II^+^CD8^+^ Tregs suppress the cytokine secretion capacity of CD4^+^CD25^−^ effector T cells (Figures 6M, N).

### MHC-II is acquired from Rapa-tolDCs by CD8^+^ Tregs via trogocytosis, with the IDO signaling pathway potentially playing a key role

**FIGURE 7:**
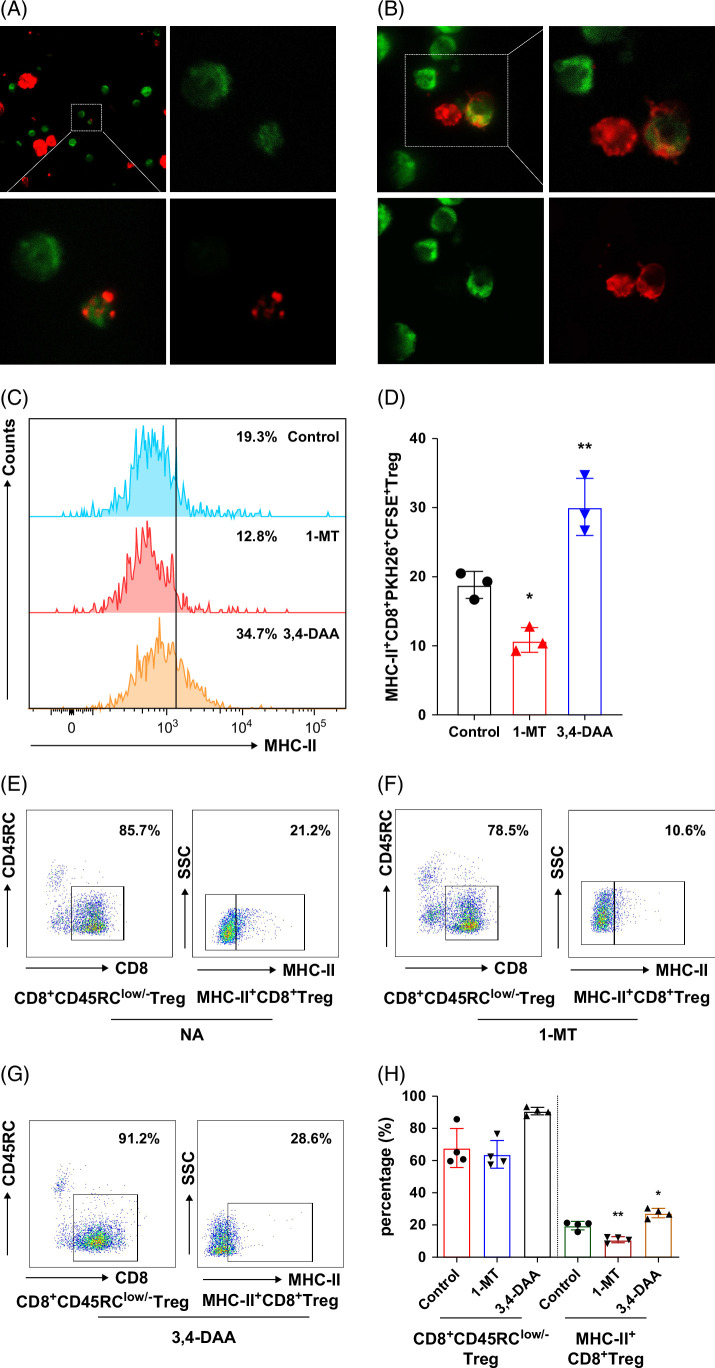
The generation mode and possible mechanism of the novel MHC-II^+^CD8^+^ Tregs induced by Rapa-tolDCs through trogocytosis. PKH26-labeled Rapa-tolDCs were co-cultured with CFSE-labeled CD8^+^ Tregs; the cells were collected on day 4 and analyzed by confocal microscopy. Confocal imaging revealed that CFSE-labeled green CD8^+^CD45RC^low/−^ Tregs surface-expressing red fluorescent-labeled cell components (A, B). The spleens were analyzed by confocal microscopy (A—upper left quadrant, original magnification ×63; others were digitally amplified to original magnification ×200). The 3,4-DAA improves the expression of MHC-II^+^CD8^+^dual-fluorescence-labeled positive cells, and 1-MT decreased according to one-way ANOVA (C, D). The fluorescence-labeling had no obvious effect on the expression of CD8+CD45RC^low/−^ Tregs and MHC-II^+^CD8^+^ Tregs by the treatment of 1-MT (E, F) and 3,4-DAA (scatter diagram (E, G), and a high level of MHC-II^+^CD8^+^ Tregs in 3,4-DAA treatment, which consist with the results of fluorescein labeling compared using one-way ANOVA (H); n=4, *p＜0.05, and **p＜0.01. Abbreviations: 1-MT, 1-methyl-tryptophan; 3, 4-DAA, 3, 4-dimethoxycinnamic acid; ANOVA, analysis of variance; CFSE, carboxyfluorescein succinimidyl ester; MHC-II, major histocompatibility complex class II; Rapa-tolDCs, rapamycin-modified tolerogenic dendritic cells; Tregs, regulatory T cells.

Rapa-tolDCs and CD8^+^CD45RC^low/−^ Tregs were labeled with red and green fluorescence using PKH26 and CFSE, respectively. After 6 days of co-culture, confocal microscopy revealed that red fluorescent fragments originating from Rapa-tolDCs were partially distributed on the surface of green fluorescent CD8^+^CD45RC^low/−^ Tregs (Figures 7A, B and Video 1). Flow cytometric analysis demonstrated that ~20% of cells exhibited dual-fluorescence (MHC-II^+^CD8^+^), consistent with previously observed proportions (Figures 7C, D). Based on these observations and prior literature, we hypothesize that trogocytosis between Rapa-tolDCs and CD8^+^CD45RC^low/−^ Tregs mediates the transfer of MHC-II molecules, resulting in the generation of chimeric MHC-II^+^CD8^+^ Tregs.

**Video 1:** After co-culture of fluorescently labeled Rapa-tolDCs and CD8+Tregs, cell components were exchanged to form new immune chimeric cells.

When the indoleamine 2,3-dioxygenase (IDO) inhibitor 1-methyl-tryptophan (1-MT) was added to the mixed culture, the number of dual-fluorescent cells per microscopic field decreased to 1–2 (Video 2). Conversely, treatment with 3,4-dimethoxyacetanilide (3,4-DAA), an IDO agonist, increased this count to 6–9 cells per field (Video 3). Flow cytometry confirmed that supplementation with 1-MT reduced, whereas 3,4-DAA enhanced, the proportion of MHC-II^+^CD8^+^ dual-fluorescent cells (Figure 7D). To exclude potential interference from fluorescent dyes, parallel experiments with 1-MT and 3,4-DAA were performed without PKH26 and CFSE labeling. Although the total proportion of CD8^+^CD45RC^low/−^ Tregs showed a nonsignificant decrease, treatment with 1-MT significantly reduced the percentage of MHC-II^+^CD8^+^ Tregs. In contrast, 3,4-DAA treatment promoted increases in both CD8^+^CD45RC^low/−^ and MHC-II^+^CD8^+^ Tregs (Figures 7E–H and Supplemental Figure S5B, http://links.lww.com/HC9/C314).

**Video 2:** 1-MT interfered with the mixed culture system of fluorescent labeled Rapa-tolDCs and CD8+Tregs, and inhibited the cell component exchange to form new immune chimeric cells.

**Video 3:** 3,4-DAA interfered with the mixed culture system of fluorescent labeled Rapa-tolDCs and CD8+Tregs, and promoted the cell component exchange to form new immune chimeric cells. The above movies can download with the following links: Links: https://pan.baidu.com/s/1VbdmiGigD8W3aeDOwY2Oww?pwd=szkh. Pass words: szkh

### The Wnt5a/Fzd4/IDO/RhoD signaling axis is essential for immunotolerance induced by consecutive Rapa-tolDC infusion through the generation of MHC-II^+^CD8^+^ Tregs

Bulk RNA sequencing data were analyzed for differential gene expression (Supplemental Figure S7A, http://links.lww.com/HC9/C314), followed by KEGG pathway analysis (Supplemental Figure S7B, http://links.lww.com/HC9/C314), Gene Ontology (GO) analysis (Supplemental Figure S7C, http://links.lww.com/HC9/C314), and GSEA–KEGG pathway enrichment (Supplemental Figure S7D, http://links.lww.com/HC9/C314). Key driver analysis (KDA) subsequently identified 563 critical genes (Supplemental Figure S7E, http://links.lww.com/HC9/C314). Clustered heatmap analysis of these differentially expressed genes revealed upregulation of Wnt5a, Wnt2, Fzd2/4, Bmp4, Axin2, GSK3β, DKK3, Kynu, Smad, IDO1, CYP1B1, and Germ1, among others, which were positively correlated with activation of the IDO1–AhR signaling pathway (Supplemental Figure S7F, http://links.lww.com/HC9/C314). These findings further substantiate the IDO-dependent signaling mechanism proposed in this study. To explore how Rapa-tolDCs regulate the transcriptional activation of IDO1 to enhance CD8^+^CD45RC^low/−^ Treg function, we examined upstream regulatory mechanisms. IDO1 is currently recognized as a downstream transcriptional target of the Wnt signaling pathway. Consistent with this, KEGG pathway enrichment analysis confirmed significant enrichment of genes associated with the Wnt signaling pathway (Supplemental Figure S7G, http://links.lww.com/HC9/C314). Collectively, these findings suggest the involvement of the Wnt/Fzd/IDO1 signaling pathway in CD8^+^CD45RC^low/−^ Tregs. However, whether Wnt2-mediated or Wnt5a-mediated signaling predominates requires further experimental validation. Trogocytosis represents a complex form of intercellular communication that may be regulated by signaling through the T-cell receptor (TCR) and Fcγ receptor (FcγR), both of which depend on the activation of small GTPases and PI3K. Based on this premise, we performed clustering analysis of differentially expressed genes involved in the TCR and FcγR signaling pathways that may participate in MHC-II molecule transfer mediated by trogocytosis. The analysis revealed upregulation of Wnt5a, Fzd4, RhoD, PIK3R3, Yes1, Gna14, Tyrobp, Fcgr1a, Mapk4, and Grip1 (Supplemental Figure S7H, I, http://links.lww.com/HC9/C314). These findings suggest that the Wnt5a/Fzd4 signaling pathway—dependent on PI3K-mediated activation of RhoD and involving Wnt5a, TDO2, PI3K, RhoD, and TC21—plays a key role in facilitating MHC-II transfer from Rapa-tolDCs to CD8^+^CD45RC^low/−^ Tregs.

**FIGURE 8:**
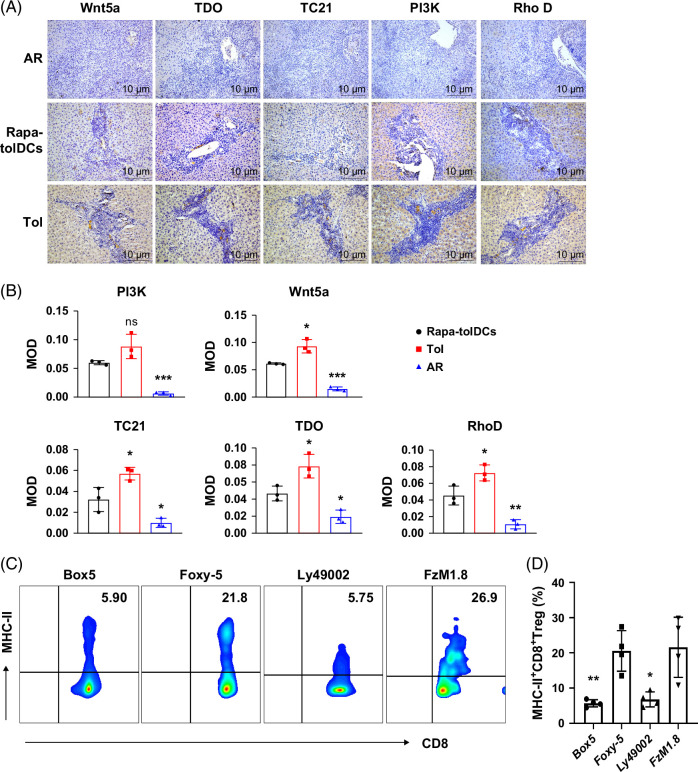
The Wnt5a/Fzd4/IDO/RhoD signal axis plays a crucial role in the MHC-II^+^CD8^+^ Tregs generation. Immunohistochemical staining was performed based on the target protein screened by RNA sequencing, which showed that Wnt5a, TDO2, RhoD, TC21, and PI3K p850 were highly expressed in the transplanted liver of rats with spontaneous tolerance and Rapa-tolDCS adoptive transfusion (A), as measured by ImageJ, indicated an obvious difference, and compared by one-way ANOVA (B). In each group of IHC experiments, n=5 tissue sections for staining, 4 independent fields for MOD determination, and the MOD average values of 3 tissue sections were selected for statistical analysis; *p＜0.05,**p＜0.01, and ***p＜0.001. It has been confirmed in Figure 7 that the IDO signaling pathway can regulate MHC-II^+^CD8^+^ Tregs; therefore, we further demonstrated the integrity of the Wnt5a/FZd4/IDO/RhoD signaling axis by using the blocking and activator of Wnt5a and PI3K. Identification of MHC-II^+^CD8^+^ Tregs was performed by flow cytometry after blocking and activating Wnt5a and PI3K when Rapa-tolDCs co-cultured with CD8^+^ T cells, compared by one-way ANOVA (C, D) (n=4). Wnt5a was activated by Box 5 and blocked by Foxy5. Fzm1.8, a steady activation of FzD4, can release the Gβγ subunit to further activate PI3K.PI3K blocked by Ly49002. Abbreviations: ANOVA, analysis of variance; IDO, indoleamine 2,3-dioxygenase; IHC, immunohistochemistry; MHC-II, major histocompatibility complex class II; MOD, mean optical density; Rapa-tolDCs, rapamycin-modified tolerogenic dendritic cells; RhoD, Ras homolog family member D; RNA, ribonucleic acid; Tregs, regulatory T cells.

This hypothesis was validated through IHC staining of liver grafts, which demonstrated that the expression levels of Wnt5a, TDO2, PI3K, RhoD, and TC21 were significantly increased in rats infused with Rapa-tolDCs, to a degree markedly higher than that observed in rats with acute rejection (Figures 8A, B). Consistent with previous findings (Figure 7), Rapa-tolDCs were shown to regulate the expression of MHC-II^+^CD8^+^ Tregs through the IDO signaling pathway, as evidenced by the effects of the IDO agonist 3,4-DAA and the inhibitor 1-MT. In co-cultures of Rapa-tolDCs with naïve CD8^+^ T cells, the addition of Box 5 and LY294002—specific inhibitors of the Wnt5a and PI3K pathways, respectively—led to a significant reduction in the proportion of MHC-II^+^CD8^+^ Tregs (Figures 8C, D). Conversely, activation of Wnt5a signaling resulted in a modest increase in MHC-II^+^CD8^+^ Treg levels (Figures 8C, D). Moreover, treatment with Fzm1.8, which activates the release of the Gβγ subunit by binding to Fzd4 and subsequently stimulates PI3K, markedly increased the number of MHC-II^+^CD8^+^ Tregs (Figures 8C, D). Collectively, these results confirm that the Wnt5a/Fzd4/IDO/RhoD signaling axis plays a pivotal role in the induction and expansion of MHC-II^+^CD8^+^ Tregs mediated by Rapa-tolDCs.

## DISCUSSION

tolDCs are regarded as a promising approach for inducing immune tolerance because they can preferentially suppress immune memory responses.^28^,^29^ However, before tolDC-based therapy can be broadly implemented in clinical settings, several major challenges must be addressed. These include developing tolDCs with stable immunomodulatory properties, optimizing infusion protocols, and elucidating the precise mechanisms by which tolDCs induce immunological tolerance.

Preclinical studies aiming to establish immune tolerance have yet to determine which subtype of tolDCs is most effective in promoting tolerance or whether the underlying mechanisms are consistent across models.^9^,^12^,^30^,^31^,^32^,^33^ Withdrawal of rapamycin (Rapa) therapy has been reported to facilitate the development of immunotolerance following liver transplantation over a period of 3 years,^34^ primarily by promoting the differentiation of tolDCs and the generation of novel memory T-cell subsets.^33^,^34^ In the present study, Rapa-induced tolDCs exhibited resistance to maturation signals triggered by LPS and enhanced the expansion of Tregs. This effect was more pronounced than that observed with tolDCs induced by vitamin D3, hormones, cyclosporine A, or mycophenolic acid.^33^

After conducting a series of experiments,^22^ we successfully established an optimized protocol that combines low concentrations of GM-CSF with small doses of rapamycin (Rapa) to generate Rapa-tolDCs. These Rapa-tolDCs exhibited enhanced immunoregulatory properties and a distinct transcriptional profile characterized by the downregulation of Siglec1 and Spp1, along with the absence of PI3K/mTOR pathway activation.

The PI3K/mTOR signaling pathway is essential for the function of other types of tolDCs generated by conventional induction methods, including those utilizing IL-10, VitD3, or hormones.^9^ In contrast, Rapa-tolDCs can circumvent inflammatory responses typically triggered by PI3K/mTOR activation. Furthermore, the reduced expression of Spp1 is closely associated with the lack of PI3K/mTOR pathway activation. We have demonstrated that the relatively low expression of Siglec1 following prestimulation represents a unique phenotype linked to tolerance induction by Rapa-tolDCs. Given the critical roles of Spp1 and Siglec1 in inducing tolDCs under conventional protocols involving IL-10 or VitD3 treatment,^9^,^32^ our findings suggest that tolerance induction by Rapa-tolDCs is independent of mTOR signaling. This may explain their selective regulation of effector and memory T-cell subsets. Collectively, these results indicate that Rapa modification addresses key limitations related to efficacy and stability in tolDC induction, offering distinct advantages that other stimulatory agents cannot achieve. Optimizing the adoptive infusion protocol to maximize the survival benefits of Rapa-tolDCs is essential for achieving immunotolerance. Understanding the mechanism through which Rapa-tolDCs induce tolerance remains a key research priority.^35^,^36^,^37^ Our previous work^22^ demonstrated that a single infusion of Rapa-tolDCs mitigated AR following liver transplantation, extending the MST to 32 days and increasing the proportion of CD8^+^CD45RC^low/−^ Tregs in the liver graft. These observations were consistent with those in sTOL rats.^18^,^22^ These findings indicate that Rapa-tolDCs promote immunotolerance primarily by inducing CD8^+^CD45RC^low/−^ Tregs. We hypothesize that the limited efficacy of a single adoptive infusion may result from an insufficient number of functional cells. To address this, we administered 3 consecutive postoperative infusions of Rapa-tolDCs, which significantly improved long-term graft survival in AR rats, extending survival to 102 days (MST 65 d). In parallel, rats that received multiple Rapa-tolDC infusions exhibited milder histopathological changes, reduced lymphocyte infiltration, lower RAI scores, and minimal fibrotic alterations compared with AR controls.

Elevated levels of MHC-II^+^CD8^+^ Tregs and their increased secretion of IL-10 in Rapa-tolDC–treated and sTOL rats were strongly associated with tolerance induction. Although MHC-II expression was also detected in AR rats, it was primarily derived from CD8^+^CD45RC^high^ T cells. This finding aligns with previous clinical reports demonstrating that elevated CD45RC expression in T cells correlates closely with allograft rejection.^38^,^39^ Furthermore, we found that MHC-II^+^CD8^+^ Tregs represented the main cellular source of IL-10 within the CD8^+^CD45RC^low/−^ Treg population. These data suggest that hypersecretion of IL-10 by MHC-II^+^CD8^+^ Tregs constitutes a crucial mechanism by which Rapa-tolDCs induce immunotolerance. However, the molecular mechanism responsible for the generation of MHC-II^+^CD8^+^ Tregs remains to be fully elucidated.

We have previously demonstrated the dual-fluorescence expression of CD8^+^CD45RC^low/−^ Tregs in cardiac grafts and spleens,^19^ and further confirmed that these MHC-II^+^CD8^+^ Tregs arise through trogocytosis-mediated acquisition of major histocompatibility complex class II (MHC-II) molecules from pDCs, thereby conferring donor-specific immune tolerance to CD8^+^CD45RC^low/−^ Tregs.^23^ Recent studies have established that trogocytosis occurs in DCs, T cells, B cells, and basophils, facilitating the intercellular transfer of MHC-II molecules and leading to the formation of novel chimeric cell subsets.^40^,^41^,^42^ In the present study, we not only verified the presence of MHC-II^+^CD8^+^ Tregs in liver grafts and spleens but also demonstrated their donor-specific suppressive function in vitro. The transfer of MHC-II molecules between Rapa-tolDCs and CD8^+^CD45RC^low/−^ Tregs via trogocytosis was clearly confirmed, supporting the authenticity and reproducibility of this phenomenon. These findings suggest that adoptive infusion of tolDCs may regulate the phenotype and functional properties of T-cell subsets by initiating trogocytosis between donor-derived and recipient-derived DCs, ultimately promoting donor-specific immune tolerance.^43^ Therefore, we propose that MHC-II^+^CD8^+^ Tregs represent a novel population of immune chimeric Tregs whose MHC-II molecules are derived from pDCs or Rapa-tolDCs.

To date, only a limited number of studies have explored trogocytosis involving DCs, CD4^+^ Tregs, CD4^+^ and CD8^+^ T cells, neutrophils, or monocytes and macrophages,^40^,^44^,^45^,^46^ with even fewer focusing specifically on interactions between CD8^+^ Tregs and DCs.^40^,^46^ The acquisition of MHC-II molecules through trogocytosis can partially confer antigen-presenting capabilities to originally non-presenting B cells or T cells.^41^,^42^ Collectively, these studies converge on the hypothesis that trogocytosis^41^,^44^ between immune cells is mediated by dual ligand–receptor interactions involving the TCR and FcγR, although the detailed molecular mechanisms remain to be elucidated.

To elucidate the underlying mechanism, we first employed fluorescein-labeled cells in a mixed lymphocyte reaction assay to confirm that MHC-II molecules present on MHC-II^+^CD8^+^ Tregs, derived from Rapa-tolDCs, can promote the generation of donor-specific MHC-II^+^CD8^+^ Tregs via the IDO signaling pathway. This conclusion was supported by the effects of the IDO agonist 3,4-dimethoxyacetanilide (3,4-DAA) and the inhibitor 1-MT.

To determine whether the IDO pathway is associated with ligand–receptor interactions, bulk RNA sequencing data revealed that activation of IDO1 during the differentiation of CD8^+^CD45RC^low/−^ Tregs was accompanied by the upregulation of Wnt5a and Fzd4—genes located upstream in the signaling cascade. The transfer of MHC-II molecules mediated by IDO from Rapa-tolDCs to CD8^+^CD45RC^low/−^ Tregs was further shown to depend on the PI3K-activated RhoD signaling pathway. Additionally, IHC staining of liver grafts from rats infused with Rapa-tolDCs or exhibiting spontaneous tolerance revealed elevated expression of Wnt5a, TDO2, PI3K, RhoD, and TC21. Inhibition of Wnt5a and PI3K signaling resulted in a pronounced reduction in the proportion of MHC-II^+^CD8^+^ Tregs. Conversely, stimulation of Fzd4 using Fzm1.8 enhanced PI3K activation, which in turn increased the number of MHC-II^+^CD8^+^ Tregs. Previous studies have suggested that in dendritic cells, IDO1 functions as a downstream transcriptional target of the Wnt signaling pathway, where Wnt5a/IDO signaling contributes to the induction of a tolerogenic phenotype.^47^ Collectively, these findings highlight the pivotal role of the Wnt5a/Fzd4/RhoD signaling axis in mediating immune tolerance through the regulation of MHC-II^+^CD8^+^ Tregs induced by Rapa-tolDCs.

This study also demonstrated that the contributions of IL-10^+^ regulatory B cells (Bregs) and Foxp3^+^ Tregs should be emphasized alongside the role of Rapa-tolDCs in tolerance induction. The induction of tolerance by Rapa-tolDCs likely involves multiple, interrelated immunoregulatory pathways. Ongoing research is investigating the mechanisms by which Rapa-tolDCs mediate tolerance through IL-10^+^ Bregs, which, together with Foxp3^+^ Tregs, appear to contribute synergistically to the establishment of immune tolerance within liver grafts. The adoptive infusion protocol for Rapa-tolDCs established in this study represents a promising approach for future immune cell therapy, potentially applicable for inducing immunological tolerance in allogeneic transplantation.^48^,^49^ This optimized protocol also provides preliminary solutions to the challenges of insufficient therapeutic cell numbers and the uncertainty surrounding optimal dosing frequency and timing in Rapa-tolDC–based adoptive cell therapy.

Collectively, our findings suggest that the adoptive infusion of Rapa-tolDCs may serve as a viable regimen for inducing donor-specific immune tolerance and supporting long-term graft survival. This effect is likely mediated by the generation of chimeric MHC-II^+^CD8^+^ Tregs through trogocytosis, regulated by the Wnt5a/Fzd4/RhoD signaling axis.

The adoptive infusion protocol for Rapa-tolDCs established in this study represents a promising approach for future immune cell therapy. It is suitable for inducing immunological tolerance in allogeneic grafts and provides an initial solution to challenges such as insufficient therapeutic cell numbers and the undetermined timing and frequency of infusion in the Rapa-tolDC adoptive infusion procedure. However, its efficacy requires further validation in large-scale experimental models. In addition, large-sample, prospective clinical studies are necessary to confirm the safety and efficacy of MHC-II^+^CD8^+^ Tregs as reliable biomarkers of tolerance. Further in-depth investigations into the molecular mechanisms by which MHC-II^+^CD8^+^ Tregs induce immunological tolerance are expected to refine the theoretical understanding of trogocytosis and tolerance induction, thereby facilitating clinical translation. Future research should also aim to determine whether chimeric MHC-II^+^CD8^+^ Tregs can be effectively generated in vitro and applied as a therapeutic infusion to establish clinically feasible immune tolerance.

## Supplementary Material
