# Supplementary material for: Rapamycin-modified novel tolerogenic dendritic cells induce liver graft tolerance through MHC-II+CD8+ regulatory T cells
Source: Hepatol Commun. 2026 Apr 17;10(5):e0942. doi: 10.1097/HC9.0000000000000942 (PMC13090084; doi:10.1097/HC9.0000000000000942)
Supplement: Supplementary file 4 [file hc9-10-e0942-s001.pdf]

Supplementary figure

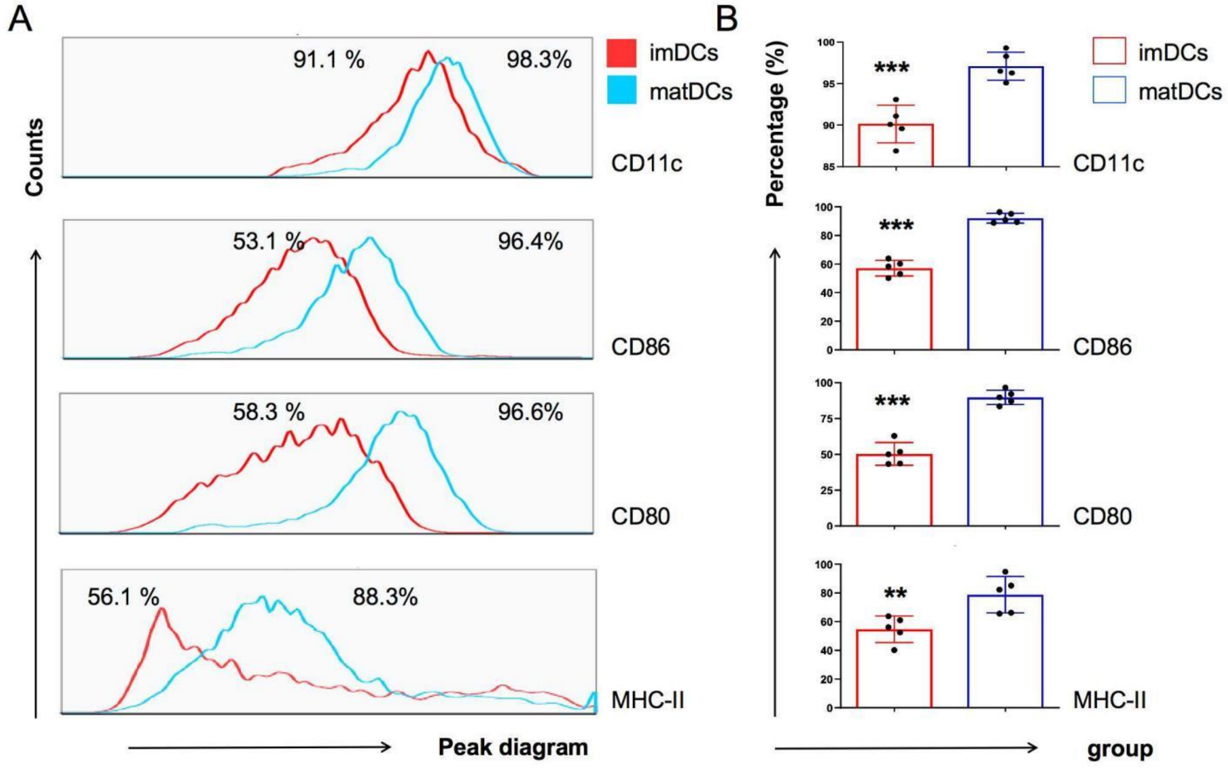

Fig. S1 High dose of GM-CSF stimulated the MSCs differentiating into imDCs and matDCs. A was the peak diagram of imDCs and matDCs with CD11c, CD80/86 and MHC-II; B was the histogram of imDCs and matDCs for CD11c, CD80/86 and MHC-II.**P＜0.01， ***P＜0.001.

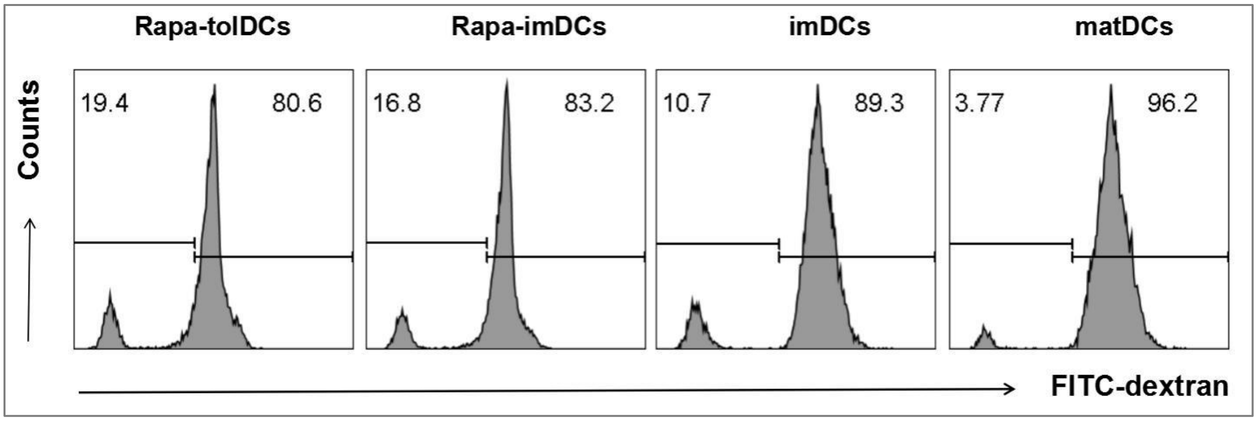

Fig S2 The representative figure of Rapa-tolDC displayed a lower taken of FITC-dextran by phagocytosis.

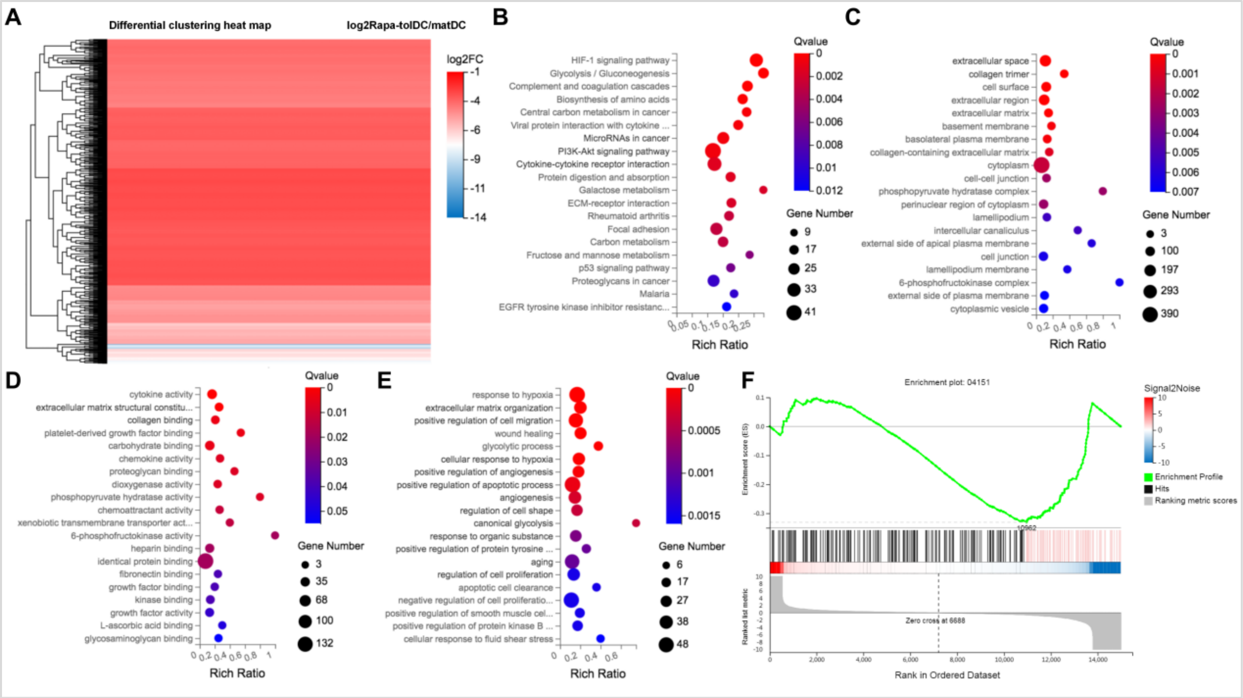

Fig. S3 Transcription profile of Rapa-tolDCs comparison with matDCs. A was the differential clustering heat map refers to log2FC of Rapa-tolDCs/matDCs which display a down-regulation of 1176 gene in Rapa-tolDCs; B, the KEGG pathway enrichment bubble chart of the differential genes which indicated 54 down-regulated or missed gene of PI3K-Akt singnal way; C to E was the GO cell component, function and process enrichment bubble chart; F, the GSEA-KEGG pathway enrichment indicated 154 down-regulated or missed gene of PI3K-Akt signal way.

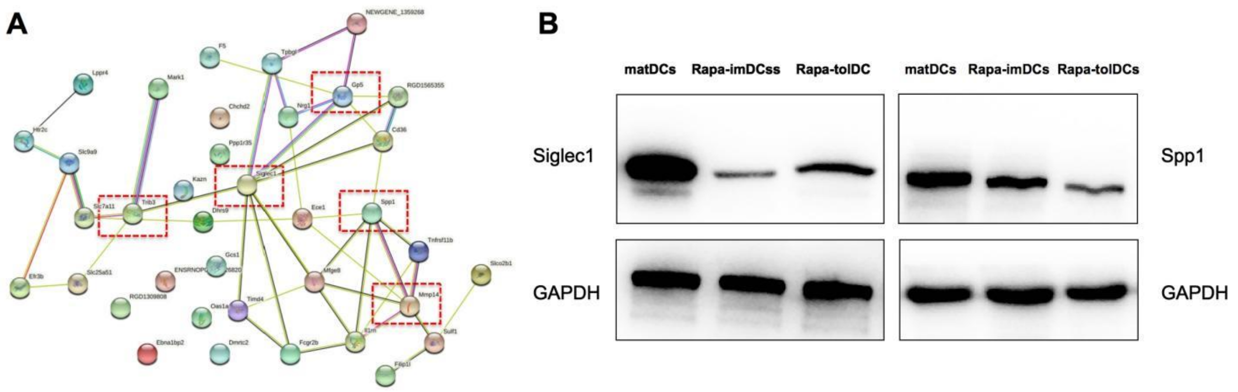

Fig S4 Expression profile analysis of key genes of Rapa-tolDCs. Protein GO enrichment analysis showed that Siglec1and Spp1,were located at key nodes (A). In the technical system of this presented study, Rapa and LPS were the two key treatment factors. Analysis of the average expression levels of the two factors in different differentiated cells showed that the expressions of Siglec1 and Spp1 after LPS treatment (matDCs) were 5824.3 and 167308.36, respectively; the expressions of Siglec1 and Spp1 after Rapa treatment (Rapa-imDCs) were 18.09 and 3629.71 and the expressions of Siglec1 and Spp1 were 539 and 2670.58 respectively after Rapa treatment and low dose LPS intervention (Rapa-tolDCs), and the differences in each group were statistically significant ($P < 0.05$). Preliminary verification of the protein level showed that Siglec1 was at a low expression level and Spp1 expression was continuously decreased, which was consistent with the results of mRNA sequencing analysis (B). The two differentially expressed genes, Siglec1 and Spp1, may be the special expression profile genes of Rapa-tolDCs.

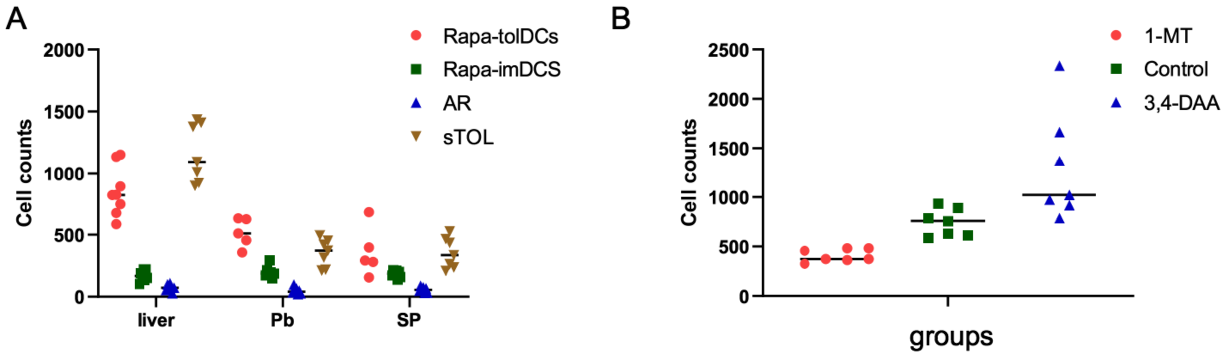

Fig S5 the absolute cell numbers in vivo (A) and intervention experiments (B)

In our pivotal experiment analyzing MHC-II$^+$CD8$^+$ Treg cells in vivo, we conducted an absolute count number analysis that showed a consistent trends with the percentage changes. The Rapa-tolDCs infusion group and sTOL group exhibited significantly elevated expression levels (Fig. S6-A). Furthermore, intervention experiments demonstrated that after 1-MT and 3,4-DAA treatments, the expression number and percentage of MHC-II$^+$CD8$^+$ Treg cells remained consistent (Fig. S6-B), with notably higher levels observed in the 3,4-DAA groups.

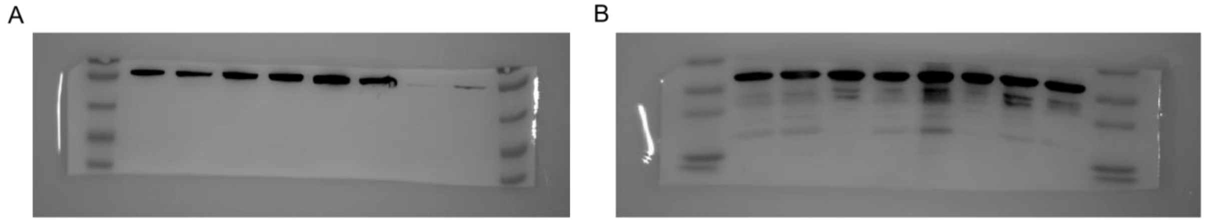

Fig S6 Uncropped of full-length blots and gels of Fig.4H for IL-10 (A) and GAPDH (B)

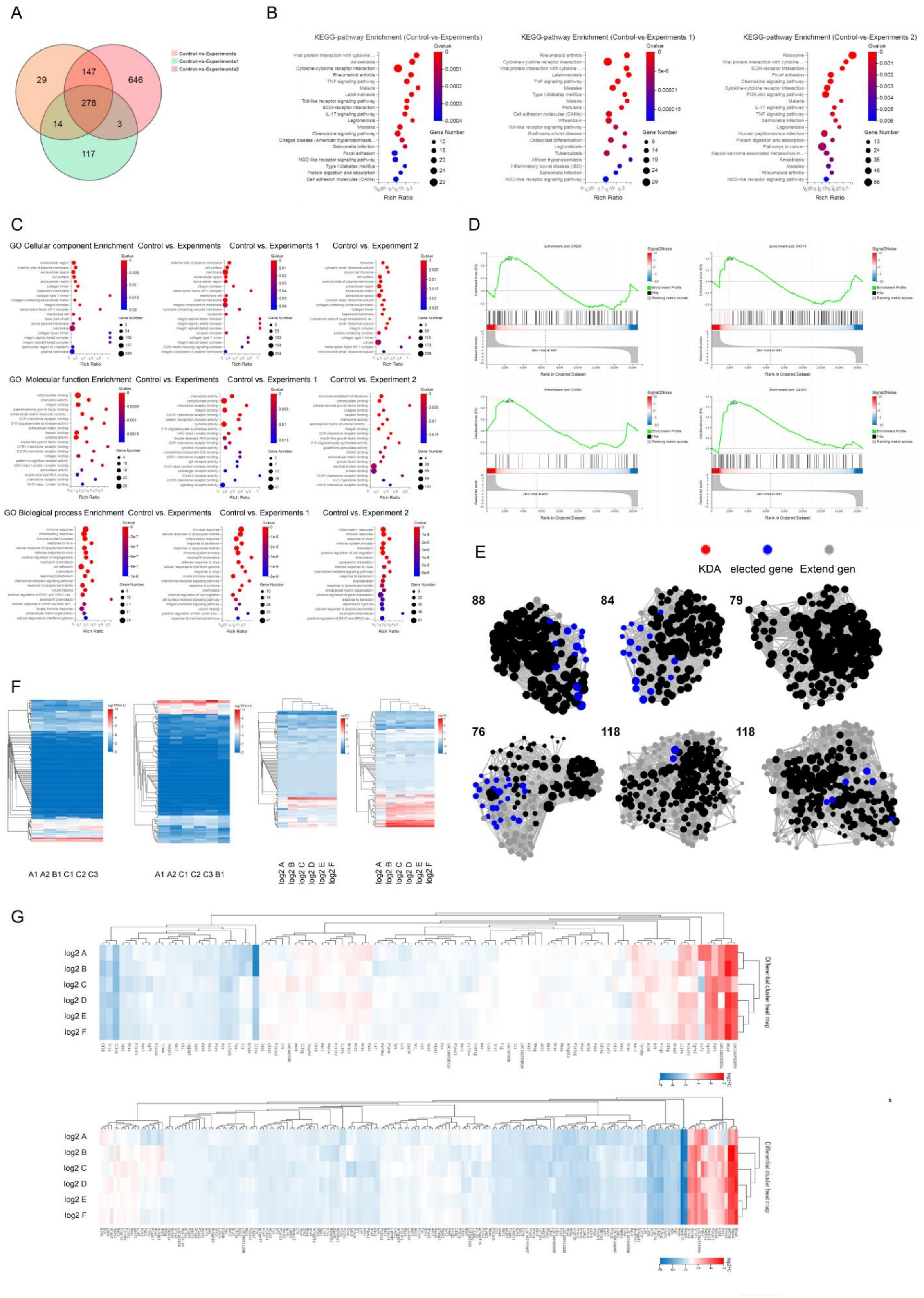

Fig S7 The RNA-Seq indicated that Wnt5a/Fzd4/IDO/RhoD signal axis plays curcial role in the MHC-II^{+}CD8^{+}Tregs generation. A total of 16,174 genes were detected by RNA sequencing, and

1234 differential genes were screened out according to different experimental groups (A). KEGG pathway enrichment of these differential genes, Mainly concentrated in the interaction between cytokines and cytokine receptors, TNF signaling pathway, Toll-like receptor signaling pathway, ECM-receptor interaction, IL-17 signaling pathway, chemokine signaling pathway, NOD-like receptor signaling pathway, cell adhesion molecules and other pathways (B); GO enrichment analysis showed that differential genes were mainly involved in the synthesis of biological process proteins such as cell membrane and extracellular region, plasma membrane and the outer part of plasma membrane, extracellular matrix, transcription factor AP-1 complex, integrin and its alpha L/M-beta2 complex, collagen (C). The Wnt, TGF-β and IDO-try-AhR signaling pathway related to regulatory T cell differentiation were enriched by GSEA-KEGG pathway (D), and 563 key differential genes were found by KDA analysis (E), and further analyzed the differential genes by clustering heat map, and found 12 genes with significant differences, including Wnt5a, Wnt2, Fzd2/4, Bmp4, Axin2, GSK3β, DKK3, Kyun, Smad, IDO1, CYP1B1, Germ1 (F). Meanwhile, the differential genes involved in TCR and FcγR signaling pathway analyzed by KDA, and then through the differential cluster analysis found that that the expression of Wnt5a, Fzd4, Rho D and Pik3r3 was enhanced in the ligand-receptor interaction pathway (G). Control,C1-C3; Experiments, A1,A2,B1; Experiments 1, A1,A2; Experiments 2, A2,B1; log2 A-F: A, Experiments/Control; B, A1,B1/C2,C3; C, Experiments1/Control; D, A1,B1/C1,C3; E, Experiments 2/Control; F, Experiments/C1,C3.

Tables

Table S1 Analysis of Rapa-tolDCs ability to stimulate PBMC proliferation

Group	OD value（450nm）	*p* value	SI	*p* value
Rapa-tolDCs	0.425±0.019		0.938±0.046	
Rapa-imDCs	0.604±0.020	0.0003	1.375±0.049	0.0024
imDCs	0.609±0.034	0.0003	1.386±0.084	0.0021
matDCs	0.713±0.039	＜0.0001	1.911±0.160	＜0.0001

OD value in 450nm was represented the proliferation effect of the PBMC after the stimulation with the inactivated Rapa-tolDCs. SI was the stimulation index.

Table S2 Phagocytosis analysis of Rapa-tolDCs

Group	FITC-dextran	*p* value	CD205	*p* value
Rapa-tolDCs	80.09±3.141		39.49±0.9831	
Rapa-imDCs	83.14±1.618	*ns*	43.50±2.565	*ns*
imDCs	88.27±1.070	0.0071	48.67±3.106	0.0325
matDCs	95.05±2.217	0.0001	64.78±4.884	＜0.0001

Video S1.

After co-culture of fluorescently labeled Rapa-tolDCs and $CD8^+$Tregs, cell components were exchanged to form new immune chimeric cells.

Video S2.

1-MT interfered with the mixed culture system of fluorescent labeled Rapa-tolDCs and $CD8^+$Tregs, and inhibited the cell component exchange to form new immune chimeric cells.

Video S3.

3,4-DAA interfered with the mixed culture system of fluorescent labeled Rapa-tolDCs and $CD8^+$Tregs, and promoted the cell component exchange to form new immune chimeric cells.

The above movies can download with the following links:
Links: https://pan.baidu.com/s/1VbdmiGigD8W3aeDOwY2Oww?pwd=szkh
Pass words: szkh
