# Supplementary material for: Rapamycin-modified novel tolerogenic dendritic cells induce liver graft tolerance through MHC-II+CD8+ regulatory T cells
Source: Hepatol Commun. 2026 Apr 17;10(5):e0942. doi: 10.1097/HC9.0000000000000942 (PMC13090084; doi:10.1097/HC9.0000000000000942)
Supplement: Supplementary file 5 [file hc9-10-e0942-s002.docx]

# **A rapamycin modified novel tolDCs induced graft tolerance of liver through MHC-II^+^CD8^+^Tregs**

Lin Zhou^1^ †*PhD, Yang Zhao^1^ † PhD, Jing Wang^2^ PhD, Qing Chen^3^ PhD, Ya-nan Jia^1^ MD, Li-chao Pan^4^ PhD , Han-xuan Wang^1^ MD, Hong-wei Yang ^5^ PhD, Qiang He^1^ PhD, Xian-liang Li^1^ PhD, Ren Lang^1^* PhD, Guo-sheng Du^1,5^ *PhD

1 Devision of Hepatobiliary and Pancreaticosplenic Surgery, Department of General Surgery, Beijing Chao-Yang Hospital, Capital Medical University, Beijing, China;100020

2 Mass General Cancer Center, Mass General Brigham, Harvard Medical School, Somerville, Massachusetts, USA; 02143

3 Department of General Surgery,Peking University Third Hospital, Beijing, China;100191

4 Faculty of Hepato-Pancreato-Biliary Surgery, Chinese PLA General Hospital, Beijing, China;100853

5 Organ transplantation center, General Hospital of Northern Theater Command, Shenyang, 110010, P.R.China

† Contributed equally

* Corresponding author

Correspondence should be addressed to,

Guo-sheng Du, Professor

E-mail: [duguosheng309@126.com](mailto:heqiang349@sina.com)

Ren Lang, Professor

E-mail: [langren@bjcyh.com](mailto:langren@bjcyh.com)

Lin Zhou, Professor

[Tel: 86-010-85231504](mailto:Tel: 86-010-85231504) E-mail: [richard_fmmu@126.com](mailto:richard_fmmu@126.com) or grandpatron@mail.ccmu.edu.cn

Address: No. 8 Gongtinan Road,Chaoyang District, Beijing, PR China,100020

## **Supplementary Methods descriptions**

**Rapa-tolDCs preparation**

The main steps of Rapa-tolDCs preparation were as follows: the extracted BM-MSCs were cultured in serum-free medium for 2 h. The suspended cells were subsequently removed and cultured in low-dose GM-CSF (10 ng/ml) with the rapamycin (10nM) regimen. The cells cultured for 6 days were named Rapa-imDCs. After the acquired Rapa-imDCs were stimulated with a small dose of lipopolysaccharide (LPS) (50ng/mL) for 24 h, the Rapa-tolDCs were obtained at 7 days.

*The molecular and immunological characteristics of Rapa-tolDCs were subsequently determined via flow cytometry and transcriptome techniques:* Monoclonal antibodies of rats' CD11c, CD86, CD80, and MHC-II were used to mark different types of DCs, see as supplementary methods of supplemental materials (SMs). The expression levels of MHC-II and costimulatory molecules on Rapa-tolDCs were determined by flow cytometry. In-taken rate of FITC-labeled dextral and expression of CD205 were adopted to identify and analyze the phagocytosis. Bulk RNA-Sequencing was used to analyzed the expression level of differential genes and transcription profile of different DCs.

*Rapa-tolDCs stimulated effector T cell proliferation assay:* Rapa-tolDCs was inactivated by mitomycin C and co-cultured with spleen derived PBMC for 48h. Optical density (OD) value was detected at 450nm and proliferation effect was calculated. The stimulation index (SI) , SI= (OD value in the experimental group - OD value in blank group) /(OD value in the control group - OD value in blank group) was also calculated .

**Adoptive infusion regimen**

The generated Rapa-tolDCs of syngeneic BN rats were separation and purification with the magnetic-activated cell sorting (MACS) (Miltenyi Biotec,USA) for adoptive transfusion of Rapa-tolDCs and Rapa-imDCs group. The Rapa-tolDCs infusion were performed by caudal vein injection, with the cell amount of 1*10^6^ each injection, and pre-stimulation 7 days before LT + three consecutive vein injections 7, 14, 28 days after LT. Meanwhile, the control group was treated with 1×PBS at each time point. The survival time and incidence of AR were recorded.

**Specimen acquisition**

The time of blood and tissue collection was set to the 7^th^ day (acute rejected rats), the 14^th^, 30^th,^ 60^th^ or beyond 90^th^ day or death (Rapa-tolDCs/imDCs infusion rats), and more than 100 days (tolerant rats).

**Mixed lymphocyte culture (MLC)**

*Rapa-tolDCs promote the differentiation of CD8^+^CD45RC^low/-^ Tregs with MLC*: After separation with MACS (Miltenyi Biotec,USA), CD8^+^ T lymphocytes with a positive ratio of >95% were mixed with Rapa-tolDCs or Rapa-imDCs for 4 days in the presence of CD3 (3 μg/ml)/CD28 (2 μg/ml) (BD bioscience, USA). Then, the supernatant was retained to further measure the level of cytokines, while the cells were collected to measure the levels of CD8^+^CD45RC^low/-^ Tregs or MHC-II^+^CD8^+^ Tregs

*IL-10 or Interferon - γ (INF-γ) was stimulated with the following protocol:* Samples were cultured for 4 h with phorbol 12-myristate 13-acetate (PMA) and ionomycin at final concentrations of 5 μg/ml and 1 μg/ml, respectively; then, monensin or brefeldin A was used for intracellular blockade. Monoclonal antibodies against CD8, CD45RC, MHC-II, TCRαβ, IL-10 and INF-γ were used to label induced MHC-II^+^CD8^+^ Tregs and secreted IL-10 and INF-γ (sites as supplementary methods of SMs). The proportion of MHC-II^+^CD8^+^ Tregs with the ability to secrete IL-10 in total induced CD8^+^CD45RC^low/-^ Tregs was analyzed to identify the subsets of cells that play major roles.

*Suppression function of MHC-II^+^CD8^+^ Tregs induced by Rapa-tolDCs:* The proliferation of 5,6- carboxyfluorescein diacetate, succinimidyl ester (CFSE)-labeled naive BN/LEW CD4^+^CD25^-^T cells cultured with or without suppressive cells at a 4:1 cell ratio for 3‒6 days at 37°C in 5% CO_2_ was detected by flow cytometry. For the donor-specific experiments, MHC-II^+^CD8^+^ Tregs were induced via incubation of recipient (BN) CD8^+^ Tregs and donor (LEW) Rapa-tolDCs. The MHC-II^+^CD8^+^ Tregs obtained by flow separation were co-cultured with CFSE-labeled recipient (BN) CD4^+^CD25^-^ effector T cells for 3 d, and mature DCs from 3rd rats (DA) were added on the 3rd day to measure donor-specific suppression. The inhibitory effect was determined by the peak size of CFSE-labeled CD4^+^CD25^-^effector T cell proliferation, refer to Li ^[20]^.

**Laser confocal microscopy scanning**

CD11^+^Rapa-tolDCs and CD8^+^CD45RC^low/-^ Tregs were separated by magnetic beads and labeled with PKH26 and CFSE, respectively. After coculture for 6 days, the molecular transfer of fluorescein-labeled Rapa-tolDCs and CD8^+^CD45RC^low/-^ Tregs was observed via confocal microscopy. Moreover, the expression levels of CFSE^+^PKH26^+^ Tregs were detected by flow cytometry.

**Signaling pathway analysis**

Rapa-tolDCs and CD8^+^ T cells were co-cultured with an IDO inhibitor (1-methyl-tryptophan, 1-MT) or analog (3,4-dimethoxy cinnamic acid, 3,4-DAA) to analyze the expression levels of CD8^+^CD45RC^low/-^ Tregs, MHC-II^+^CD8^+^ Tregs and IL-10, respectively. The fluorescein-labeled protocol was consistent with the protocols listed in the supplementary methods of SMs. Bulk RNA sequencing was used to analyze the expression levels of differentially expressed genes, and the pathway of the generation of MHC-II^+^CD8^+^ Tregs. Immunohistochemistry was also used to verify the expression of differentially expressed genes in liver grafts. Blockade and activation of Wnt5a and PI3K were further used to analyze the signaling pathway of MHC-II^+^CD8^+^ Tregs.

**Flow cytometry analysis**

CD8^+^CD45RC^low/-^ Tregs, MHC-II^+^CD8^+^ Tregs, IL-10 and INF-γ in the peripheral blood, grafts and spleen of model rats and cultured cells were detected via flow cytometry.

The sample was first supplemented with an appropriate amount of monoclonal antibody, incubated for 15 min in the dark, and then washed with 1× PBS for detection. For intranuclear cytokines, membrane breakage should be continued, followed by incubation with antibodies, washing with 1× PBS, and preparation for detection. The detailed steps and labeling protocol for the fluorescent antibodies are shown in the supplementary methods of SMs.

**HE staining and immunohistochemistry**

After being fixed in formalin, dehydrated, embedded, sectioned to a thickness of 5 μm, and stained with hematoxylin‒eosin (HE) and Masson to measure the AR of liver graft.

The immunohistochemistry (IHC) staining of Wnt5a, TDO2, TC21 Rho D, and PI3K p85β and LC3 and P62 was performed as follows: the 10% formalin-fixed tissues were embedded in paraffin, sectioned to 5 μm with a microtome and heated at 60°C on slides for 30 min before they were dewaxed, inactivated by 3% H2O2 inactivation, pretreated in a microwave at 100°C for 20 min and incubated with a closed antibody for 30 min. The disposed tissues were incubated with primary antibodies against Wnt5a, TDO2, TC21 Rho D, PI3K p85β and LC3 (1:100, Abcam, USA) , P62 (1:1500, Bioss, China)；overnight and then incubated with a second antibody against PV-9000 kits (ZSGB-BIO, Abcam, USA) for 60 min. The results of the IHC staining were detected with a Nikon microscope system (Germany) and a DAB kit (ZSGB-BIO, USA).

**Protein isolation and Western blotting**

Total protein was prepared by trypsinization of liver cells isolated from grafts in 4°C lysis buffer. The purity of the protein was subsequently determined via a BCA assay according to the manufacturer’s instructions. Western blotting was conducted in the following order: preparation of a separation gel and samples, electrophoresis, membrane transfer, blocking, antibody incubation, development, and fixation.

**Multiplex immunofluorescence assay**

Donor-specific CD8^+^Tregs in liver grafts subjected to multiplex immunofluorescence staining (mIFs) were stained with an Opal 7 color manual IHC kit (PerkinElmer, Hopkinton, MA, USA). The steps before incubation with the primary antibodies were the same as those used for the above mentioned IHC staining. Tissues were incubated with antibodies against CD8, CD45RC, MHC-II and IL-10 for MHC-II^+^CD8^+^Treg, CD5, CD1d, and IL-10 for IL-10^+^Breg and CD4, Foxp3 for CD4^+^Foxp3^+^Treg, serially for 1 hour and further incubated with the corresponding Opal fluorophore for 10 minutes to visualize the combined antibodies. Then, the tissue sections were labeled with anti-rabbit/mouse polymeric horseradish peroxidase (PerkinElmer, Hopkinton, MA, USA) for 10 minutes. Finally, the sections were counterstained with spectral DAPI for 10 minutes, mounted with anti-fade mounting medium (P36965, Life Technologies) and stored at 4°C before they were scanned with the Vectra Polaris multispectral imaging platform (Akoya Biosciences /OLYMPUS) , during which 5–7 images of interesting regions were acquired for further analysis. Image analysis was performed via In Form 2.4.8 Image Analysis Software (Akoya Biosciences/OLYMPUS).
